# Can Pakistan achieve the UNGA 70% antibiotic access target? A scoping review of susceptibility data

**DOI:** 10.3389/fpubh.2026.1778876

**Published:** 2026-05-01

**Authors:** Fiza Benish, Zikria Saleem, Shairyar Afzal, Huda Arooj, Mahmoud E. Elrggal, Abdul Haseeb

**Affiliations:** 1 Department of Pharmacy Practice, Faculty of Pharmacy, Bahauddin Zakariya University, Multan, Punjab, Pakistan; 2 Department of Pharmacy Practice, College of Pharmacy, Qassim University, Buraydah, Saudi Arabia; 3 Department of Pharmacy, DHQ Hospital Jhelum, Jhelum, Pakistan; 4 College of Medicine, Al Qunfudah Umm Al-Qura University, Makkah, Saudi Arabia; 5 Department of Pharmacy Practice, Faculty of Pharmacy, University of Tabuk, Tabuk, Saudi Arabia

**Keywords:** access antibiotics, antibiotic agent, antibiotic resistance, antibiotics, antimicrobial resistance, antimicrobial susceptibility, WHO AWaRe antibiotics

## Abstract

**Background:**

Antimicrobial resistance (AMR) poses a major public health challenge in Pakistan due to irrational antibiotic use and weak surveillance. The WHO AWaRe framework and United Nations General Assembly (UNGA) declaration urge a 70% global use of Access antibiotics. This scoping review aims to assess whether current susceptibility patterns in Pakistan (2020–2024) support achieving or maintaining the 70% Access target.

**Methods:**

A PRISMA-ScR–based scoping review identified 2020–2024 studies reporting susceptibility of WHO Access antibiotics in Pakistan. Eligible studies included human clinical isolates tested by standardized antimicrobial susceptibility methods. Weighted pathogen-antibiotic-year-wise susceptibilities were analyzed, along with pathogen-and WHO ATC (anatomical therapeutic chemical classification) class–wise temporal trends.

**Results:**

A total of 74 studies published between 2020 and 2024 were included. Most studies were conducted in Punjab. Overall, the susceptibility of WHO Access antibiotics remained low across major pathogens. *S. aureus* and Pseudomonas spp showed notable declines, while *E. coli* has shown variable susceptibility. Among WHO ATC classes, aminoglycosides, beta lactams, and cephalosporins demonstrated low susceptibility.

**Conclusion:**

This scoping review revealed that persistently low susceptibility to Access antibiotics in Pakistan challenges the feasibility of the WHO's 70% utilization target. Strengthening surveillance programs is essential to curb AMR and align national efforts with global antibiotic use targets.

## Introduction

Antimicrobial resistance (AMR) has emerged as one of the most critical threats to global public health, leading to prolonged illness, increased mortality, and growing healthcare costs worldwide (1–5). A recently published Global Burden of Disease (GBD) Study reported that 4.71 million deaths were associated to bacterial AMR in 2021, encompassing 1.14 million directly attributable to bacterial AMR (6). The burden is disproportionately high in low- and middle-income countries (LMICs), including Pakistan, where factors such as unregulated antibiotic sales and over-the-counter dispensing in the community, self-medication, and weak stewardship infrastructure accelerate the spread of resistant pathogens (7, 8). Despite being considered essential first-line therapies, even the most commonly used antibiotics are now at risk of losing efficacy due to inappropriate prescribing practices, empirical therapy without culture support, and poor infection control measures (9).

To address this global threat, the World Health Organization (WHO) introduced the AWaRe (Access, Watch, Reserve) classification of antibiotics in 2017. This periodically updated classification categorizes antibiotics into Access, Watch, and Reserve groups based on their resistance potential, cost, and ease of availability. The Access group is considered the first line for numerous infectious diseases presented to primary care and hospital facilities (10). This group includes narrow-spectrum agents from antibiotic classes prioritized for their lower potential to drive resistance and greater affordability, making them an excellent choice against common infections, and simultaneously calling for their rational use, which is crucial, especially in LMICs (11, 12).

In Pakistan, inappropriate antibiotic use remains a growing concern across both hospital and community settings. Qualitative studies exploring physicians' perceptions have identified notable gaps in knowledge, inadequate diagnostic support, unclear local guidelines, and external pressures such as pharmaceutical marketing, all of which drive irrational prescribing behaviors (13). As a result, the consumption of Watch-category antibiotics has markedly increased. Studies have reported that between 59.5 and 72% of all prescribed antibiotics belong to the Watch group, substantially exceeding the recommended use of Access agents (13–16). This trend is alarming, as the overuse and misuse of both Watch and Access antibiotics have been frequently observed, contributing to the growing resistance among key pathogens such as *Escherichia coli, Staphylococcus aureus*, and *Pseudomonas* spp (17–20). In line with these concerns, high resistance has been reported among *E. coli* and *Klebsiella* isolates from pediatric urinary tract infections, where Access agents showed poor activity and last-line agents such as carbapenems and colistin remained effective (21, 22).

In response to the rising burden of AMR, Pakistan launched its national action plan on AMR (NAP-AMR) (23) in alignment with the WHO Global Action Plan in May 2017 (11). The NAP emphasizes strengthening surveillance systems, regulating antimicrobial use, and improving stewardship practices across human, animal, and environmental health sectors. However, despite these policy efforts, NAP-AMR suffers from implementation bottlenecks (8). Furthermore, there is a lack of continuous structured surveillance of AMR, particularly resistance among microorganisms previously susceptible to Access group antibiotics (24, 25). Following the Global Action Plan to tackle antimicrobial resistance at the 68th World Health Assembly, 2015, the global antimicrobial resistance surveillance system (GLASS) was established to collate antimicrobial resistance data at a global level (26). There is a critical need for updated, evidence-based assessments of resistance patterns against Access antibiotics to inform clinical guidelines and support the objectives of the NAP-AMR (23). Additionally, the United Nations General Assembly (UNGA) high-level meeting on AMR in September 2024 recommended 70% Access antibiotics utilization globally. In line with the UNGA recommendation, it is important to evaluate the susceptibility pattern of Access antibiotics in Pakistan. The 2024 UNGA Political Declaration reaffirmed AMR as a pressing global health challenge and underscored the importance of strengthening surveillance of Access antibiotics in high-burden countries such as Pakistan (27).

Although multiple isolated studies in Pakistan have reported on antibiotic susceptibility, there is a lack of comprehensive, time-bound, and stratified analysis focused specifically on Access group antibiotics. Without this, it remains difficult to track susceptibility trends over time, assess inter-pathogen variability, or understand resistance dynamics at the pharmacological class level (2, 28, 29).

This scoping review aims to fill that gap by synthesizing published evidence from 2020 to 2024 on susceptibility patterns of Access group antibiotics in Pakistan. The review evaluates year-wise changes, pathogen-specific susceptibility patterns, and pharmacological class-level trends. These findings can support data-driven prescribing, inform antimicrobial stewardship policies, and contribute to Pakistan's response to the global AMR crisis.

## Methodology

A scoping review was conducted to evaluate publications on susceptibility patterns of Access group antibiotics in Pakistan between 2020 and 2024. This review was designed and reported following the PRISMA-ScR (preferred reporting items for systematic reviews and meta-analyses extension for scoping reviews) checklist. The objective, search strategy, and study selection criteria were pre-specified according to a documented protocol.

This study utilized data from peer-reviewed articles reporting susceptibility patterns of commonly used access antibiotics against frequently isolated pathogens. For each pathogen-antibiotic-year combination, susceptibility percentages were adjusted for the number of isolates tested, yielding weighted susceptibility values. Year-wise weighted averages were then computed to assess susceptibility trends at both the pathogen level and at the WHO ATC class level. In the anatomical therapeutic chemical (ATC) classification system, The active ingredients are distinguished into various groups based on their chemical, pharmacological, and therapeutic characteristics as well as the organ or system they work on (30).

Antibiotics were grouped into their respective pharmacological classes (e.g., Beta-lactams, Aminoglycosides, Sulfonamides), and aggregate class-wise susceptibilities were calculated annually to understand broader therapeutic performance.

### Eligibility criteria

This review followed the population-concept-context (PCC) framework as recommended by the Joanna Briggs Institute for scoping reviews (31). The selection criteria were designed to be broad and inclusive, accounting for the anticipated scarcity of literature on this topic, while also incorporating gray literature and reference tracking for comprehensiveness.

### Inclusion criteria

- English-language publications were included, as English is the dominant scientific language used in Pakistan.
- Original quantitative studies were selected that reported antibiotic susceptibility or resistance patterns in Pakistan.
- Studies that included at least one Access group antibiotic (as classified by the WHO AWaRe system) in their susceptibility profiles.
- Studies involving human clinical isolates collected from hospital or laboratory settings.
- Research conducted in diagnostic laboratories with clearly defined antimicrobial susceptibility testing (AST) methods and interpretive criteria.
- Articles that reported the total number of isolates tested, along with the percentage of resistant and susceptible organisms.

**Population:** Patients attending outpatient departments, inpatient departments, and those whose samples were processed in diagnostic laboratories or hospitals, including regional healthcare facilities across Pakistan.

**Concept:** Resistance, susceptibility, or sensitivity patterns of pathogens such as Escherichia coli, Staphylococcus aureus, and multi-drug resistant (MDR) bacteria, with a specific focus on Access group antibiotics.

**Context:** Secondary and Tertiary care hospitals (TCHs), diagnostic centers, and clinical research laboratories based in Pakistan.

### Exclusion criteria

- Studies involving microorganisms of environmental, poultry, or animal origin.
- Publications prior to 2020.
- Studies published in languages other than English.
- Studies that did not include Access group antibiotics in their susceptibility profiles.
- Studies reporting data from countries other than Pakistan and multi-country data, including Pakistan.

### Statistical approach

Descriptive statistical method was used to assess trends in antimicrobial susceptibility over time (32). To account for varying isolate counts across antibiotics and years, a weighted susceptibility percentage was calculated for each pathogen-antibiotic-year combination by using the following formula:

Weighted Susceptibility (Antibiotic) = (No. of Susceptible Isolates / Total Isolates) × 100

Subsequently, weighted averages were computed at the pathogen level to assess the overall susceptibility of each common organism per year, considering all antibiotics tested against it. This allowed for temporal comparison of susceptibility patterns across years. Used the following formula:

Weighted Avg. Susceptibility (Pathogen) = ∑ (Weighted Susceptibility_i_ × Total Isolates_i_) / ∑ Total Isolates_i_

In addition, antibiotics were grouped into pharmacological classes (e.g., Beta-lactams, Aminoglycosides, Sulfonamides), and class-wise weighted susceptibility was calculated per year to evaluate broader therapeutic class performance over time by using the following formula:

Weighted Avg. Susceptibility (class) = ∑ (Weighted Susceptibility_i_ × Total Isolates_i_) / ∑ Total Isolates_i_.

### Recognizing and generating research questions

This review mainly concentrated on the susceptibility patterns of Access group antibiotics against various microbes in Pakistan, which is essential for guiding effective treatment strategies and informing public health policy. After a detailed initial review of the literature, the following sub-research questions (RQs) were formulated:

- **RQ1:** What are the temporal trends in antibiotic susceptibility of Access group antibiotics across Pakistan from 2020 to 2024?
- **RQ2:** How do *Escherichia coli, Staphylococcus aureus*, and *Pseudomonas spp*. differ in their susceptibility patterns to Access group antibiotics over the study period?
- **RQ3:** Which pharmacological classes of Access group antibiotics (as per WHO anatomical therapeutic chemical (ATC) classification show improvement or decline in susceptibility trends from 2020 to 2024?

### Search strategy and study selection

Three bibliographic databases, namely Google Scholar, PubMed, and ScienceDirect, were searched by employing relevant keywords and index terms to identify research papers between January 2020 and December 2024.

The keywords utilized in the search strategy followed a combination of multiple string terms separated by Boolean operators ‘AND' or ‘OR'. The first-string term included “antimicrobial resistance”, “antibiotic resistance”, “drug resistance”, “AMR” and “antibiotic susceptibility”. The second string comprised “WHO AWaRe classification”, “AWaRe”, “Access Watch Reserve” and “WHO Access antibiotic”. The third term included “health care facility”, “hospital”, “public hospital”, and “healthcare setting”. These terms were further combined with geographical identifiers, “Pakistan” or “Pakistani”.

Titles and abstracts of potentially pertinent studies were used to find them. Furthermore, all pertinent research was thoroughly studied and chosen based on the inclusion criteria.

### Identification and screening of articles

**Figure 1:**
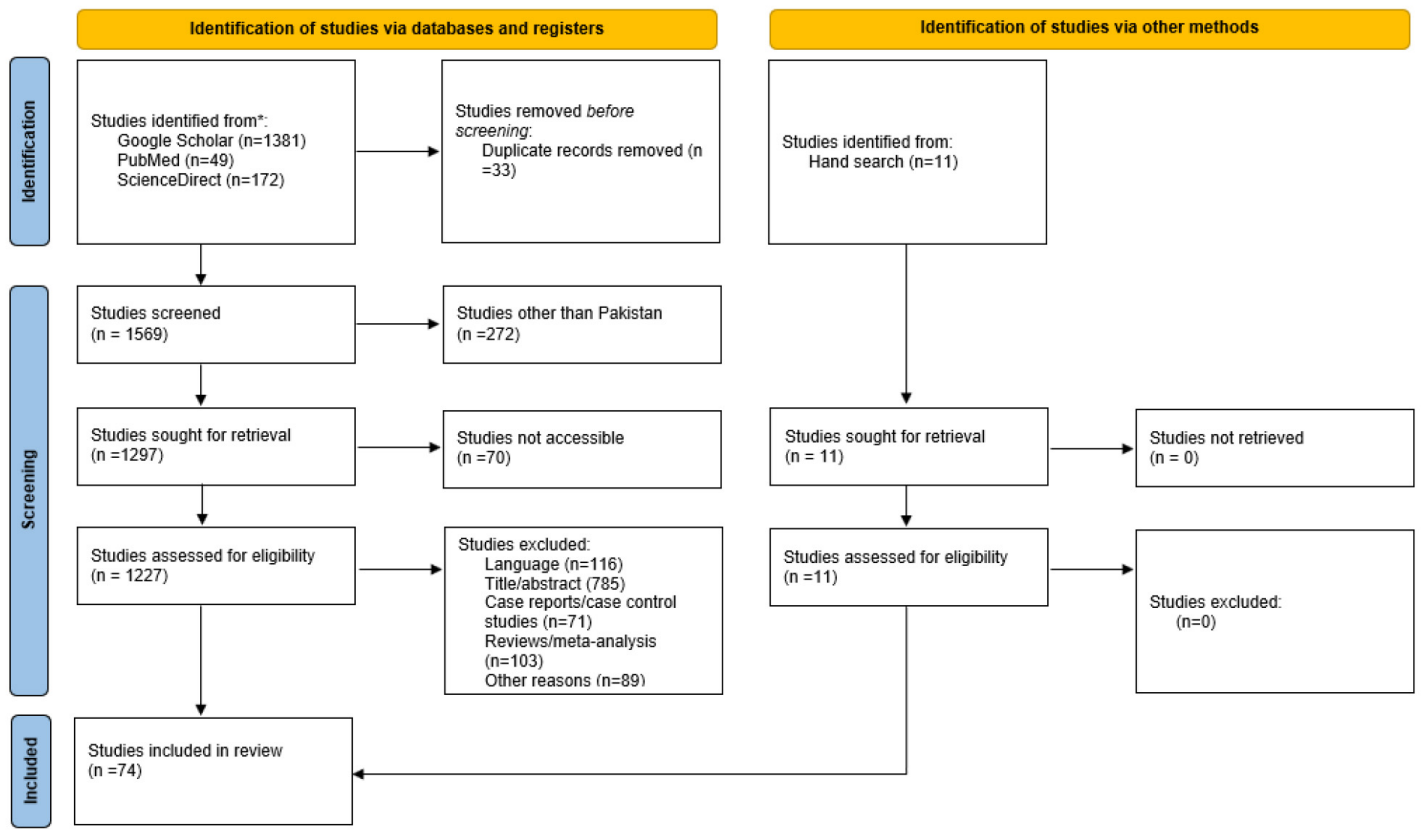
PRISMA flow chart showing exclusion and inclusion of studies.

A total of 1,613 published articles were identified via databases and hand search. After the removal of duplicates, a total of 1,580 articles were identified. Only 74 articles met the inclusion criteria and were included in this study. The remaining 1,539 articles did not meet the requirements for inclusion as they did not include any access group antibiotic, did not include a proper antibiotic resistant or susceptibility profile, were not conducted in Pakistan, were not in the English language, the study was a review, an editorial, a correspondence, or a case report (Figure 1) (33). The screening of the articles were performed independently by two researchers (FB) and (SA). Any difference of opinion regarding the inclusion of the studies was resolved through mutual discussion in the presence of the project leaders.

### Data extraction and charting

Data extraction and analysis were conducted following the PRISMA-ScR (preferred reporting items for systematic reviews and meta-analyses extension for scoping reviews) guidelines. Key attributes were extracted using a pre-designed data collection form. These included the first author, year of publication, region/city of study, study duration, healthcare setting, type of disease, sample size and site, population, type of isolated microorganism, study design, and key findings, including susceptibility results of Access group antibiotics against various microorganisms.

Extracted data were subsequently classified into thematic categories aligned with the objectives of the study:

Category **1**: Temporal Distribution of Resistance Studies in Pakistan Category **2**: Year-Wise Weighted Susceptibility of Access Group Antibiotics Category **3**: Pathogen-Specific Susceptibility Patterns Category **4**: Antibiotic Class-Wise Resistance Profiles.

Where applicable, individual antibiotic susceptibility percentages were classified according to the WHO AWaRe classification (Access, Watch, Reserve), and cumulative percentages were computed. Most results were reported as presented in the original studies without modification. In instances where studies reported resistance percentages only, these were converted to susceptibility values. Similarly, when only the number of resistant and total isolates was provided, susceptibility percentages were calculated directly to ensure consistency across datasets.

## Results

### Literature features

Among all the publications gathered from search engines using different keywords, 74 were chosen for final analysis based on the inclusion and exclusion criteria. Out of 74 studies, 38 studies had information about both gram-negative and gram-positive isolates (34–71), 30 studies were from gram-negative isolates (67, 72–100) and 6 had information about gram-positive isolates (101–106).

**Figure 2:**
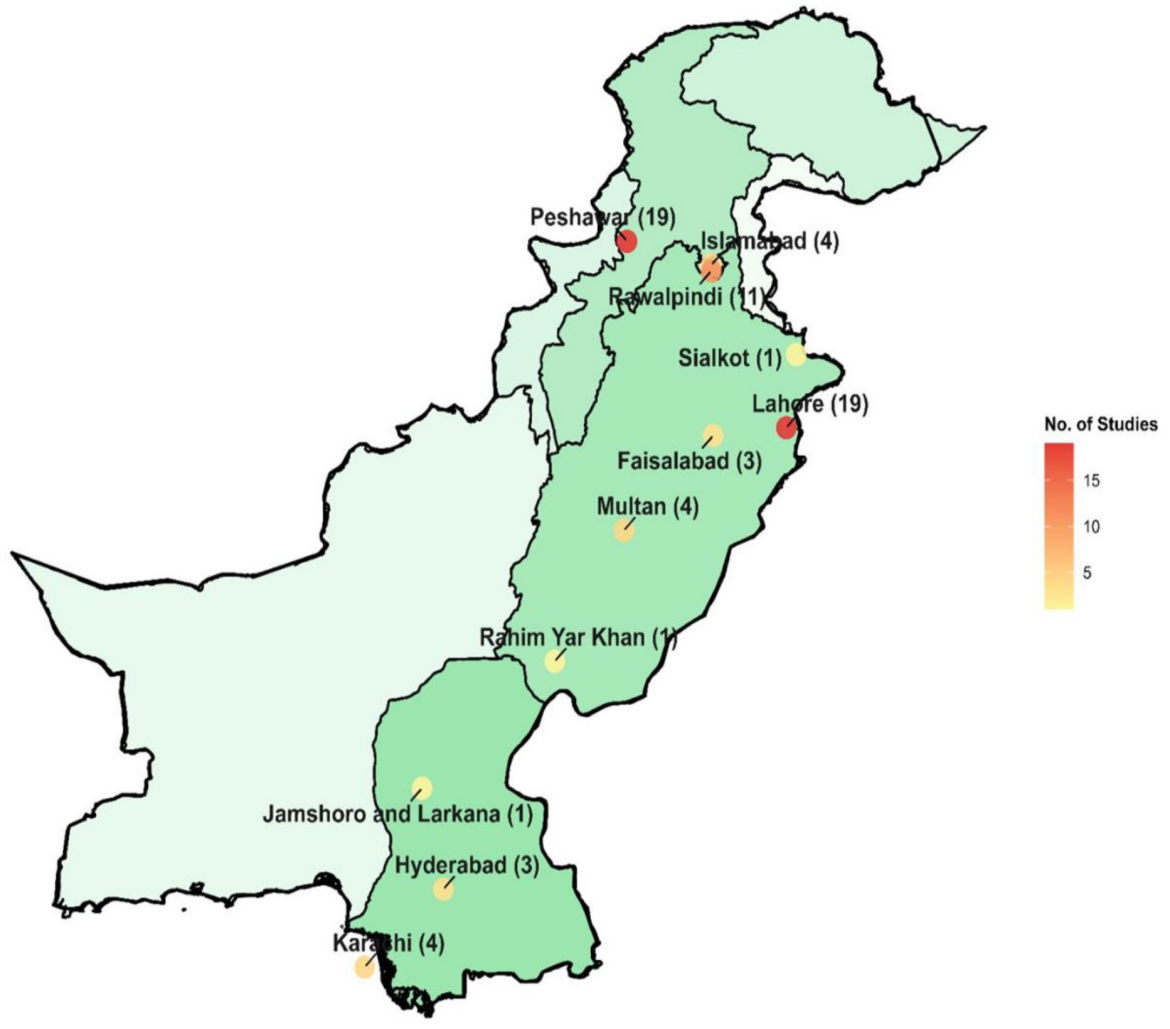
Number of antibiotic-susceptible studies from different cities of Pakistan included in this study.

There are four provinces in Pakistan and the capital territory, i-e, Islamabad. The majority of the studies (25.67%) occurred in Lahore (Punjab) and Peshawar (KPK), 22.97%. While 14.86% of the studies were from Rawalpindi city. Two research studies from Punjab province did not specify the name of the city (68, 87) and another two studies from Khyber Pakhtunkhwa (KPK) province and the district Swat also didn't mention the name of the city (Figure 2) (52, 89).

The maximum number of studies was specified in 2023 (35.13%), followed by 2020 (25.67%), 2022 (24.32%), 2021 (10.81%), and 2024 (4.05%). In 10 studies, the duration of the study was not mentioned (67, 76, 80, 82, 84, 87, 89, 93, 99, 104). Phenotypic and genotypic evaluation of antibiotic resistance was mentioned in two studies (79, 89). Out of all the research, 20 (27.02%) had UTI as the most frequently documented clinical diagnosis. Based on data related to UTI, one study mentioned the community-acquired UTI (63). However, in 19 (25.67%) studies, infection type was not specified (44, 45, 48, 57, 61, 67, 68, 71, 79, 80, 83, 93, 95, 97, 98, 100, 102, 104, 105). The distribution of studies by patient type, gender, age groups of Gram-positive and Gram-negative isolates, and type of clinical samples is summarized in Supplementary Table S1. Main findings and detailed characteristics of the included studies from 2020 to 2024 are provided in Supplementary Table S2, while information on common microorganisms and Access group antibiotics reported across these studies is summarized in Supplementary Table S3.

### Weighted antibiotic susceptibility by year

**Figure 3:**
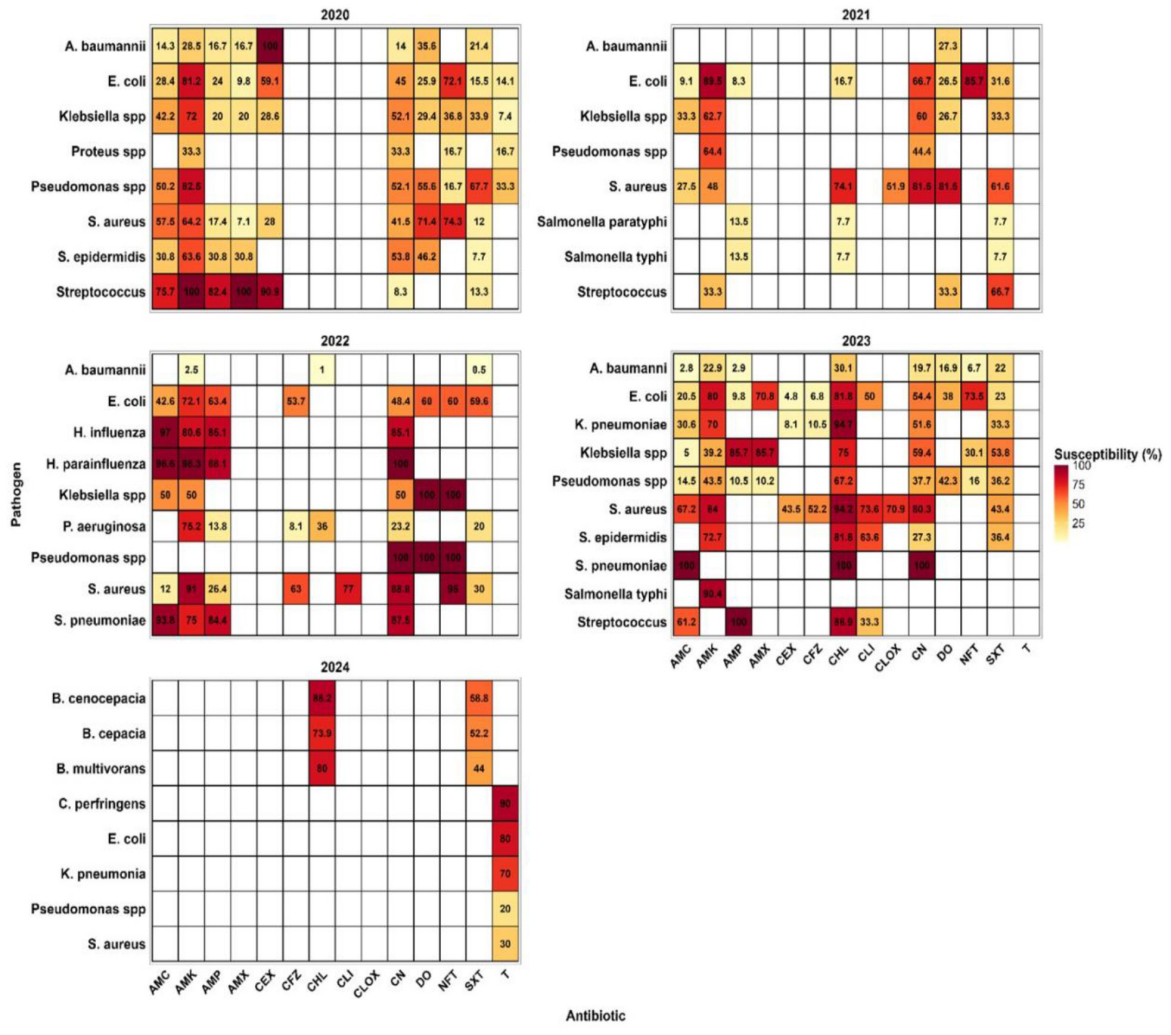
Weighted antibiotic susceptibility by year for different pathogens against Access group antibiotics from 2020 – 2024. AMC, amoxicillin–clavulanate; AMK, amikacin; AMP, ampicillin; AMX, amoxicillin; CEX, cephalexin; CFZ, cefazolin; CHL, chloramphenicol; CLI, clindamycin; CLOX, cloxacillin; CN, gentamicin; DO, doxycycline; NFT, nitrofurantoin; SXT, trimethoprim–sulfamethoxazole (co-trimoxazole); T, trimethoprim.

A weighted average susceptibility was calculated for each pathogen–antibiotic–year combination to standardize results across multiple studies and isolate counts. Of isolates reported between 2020 and 2024, susceptibility to WHO Access-group antibiotics varied considerably across pathogens (Figure 3). For *Escherichia coli*, susceptibility to ampicillin ranged from 8.3 to 63.4%, with the highest value observed in 2022, while co-trimoxazole ranged from 15.5 to 59.6%. By contrast, amikacin consistently exceeded the threshold across all years (72.1%−89.5%), and nitrofurantoin also demonstrated relatively high activity (60.0%−85.7%).

*Klebsiella spp*. showed weaker performance, with amoxicillin-clavulanate and ampicillin ranging from 5.0 to 50.0%, although an isolated higher value for ampicillin (85.7%) was observed in 2023. Amikacin exhibited only moderate activity (39.2%−72.0%). *Acinetobacter baumannii* showed markedly reduced activity to amikacin, declining to 2.5% in 2022, while *Pseudomonas spp*. demonstrated decreasing susceptibility over time (82.5% in 2020, 64.4% in 2021, and 43.5% in 2023).

Among Gram-positive organisms, *Staphylococcus aureus* displayed variable resistance patterns. Susceptibility to ampicillin and amoxicillin-clavulanate was low (e.g., 26.4% to AMP in 2022), whereas amikacin consistently showed high activity (84.0%−91.0% in 2022–2023). Chloramphenicol also emerged as a highly active agent, with susceptibility reaching 94.2% in 2023. Among Gram-negative respiratory pathogens (*Haemophilus influenzae* and *H. parainfluenzae*), reported in 2022, demonstrated uniformly high susceptibility (>80%) to both amoxicillin-clavulanate and amikacin.

Taken together, these findings confirm that most Access antibiotics, particularly aminopenicillins and co-trimoxazole, showed poor activity against Gram-negative organisms and variable performance against Gram-positives. Notable exceptions included amikacin for *E. coli* and *S. aureus*, and aminopenicillins for respiratory pathogens. These results underscore the inadequacy of relying solely on Access agents for empirical therapy in this setting and highlight the importance of local AMR surveillance to inform stewardship policies.

### Interpretation of pathogen-wise susceptibility percentages

**Figure 4:**
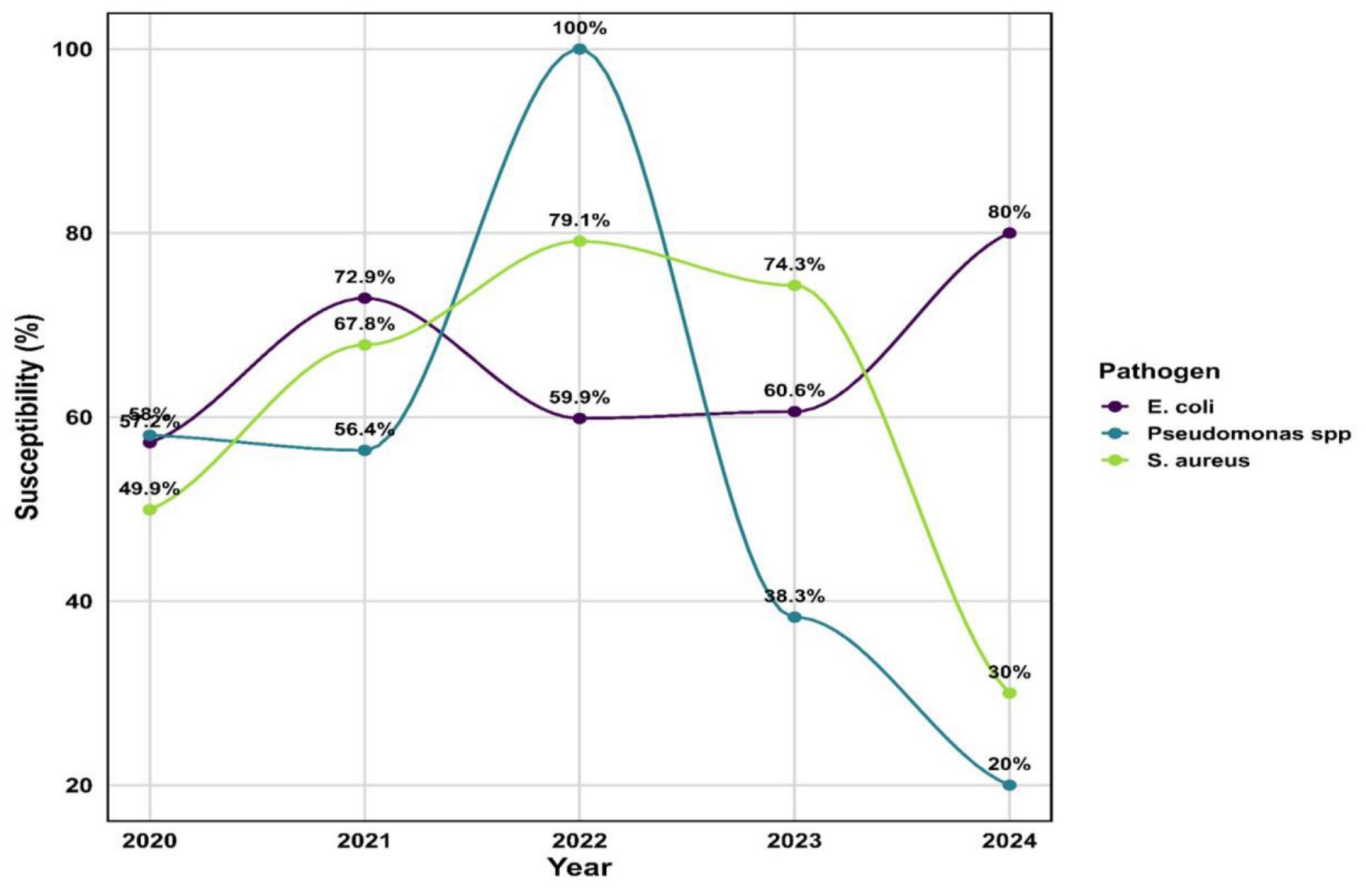
Year-wise weighted susceptibility trends of common pathogens (E. coli, Pseudomonas spp., Staphylococcus aureus) from 2020 to 2024.

Of pathogens reported across 2020 – 2024, only *Escherichia coli, Pseudomonas* spp, and *Staphylococcus aureus* were consistently represented in all five years, allowing for weighted susceptibility trend analysis (Figure 4).

For *S. aureus*, susceptibility improved steadily, reaching 79.1% in 2022, but declined sharply to 30% in 2024, a finding likely influenced by the reduced number of isolates tested. For *E. coli*, susceptibility remained moderate and relatively stable (57%−60%) across most years, with a higher value of 80% observed in 2024, again attributable to the small sample size. For *Pseudomonas* spp, susceptibility showed greater variability, with values declining markedly to 20% in 2024.

### Interpretation of class-wise susceptibility percentages

To evaluate antibiotic susceptibility over time, we grouped individual antibiotics into their respective WHO ATC classes and analyzed weighted susceptibility percentages year-wise (107). This approach allowed us to track resistance patterns at a broader, pharmacological class level rather than focusing solely on individual agents.

**Figure 5:**
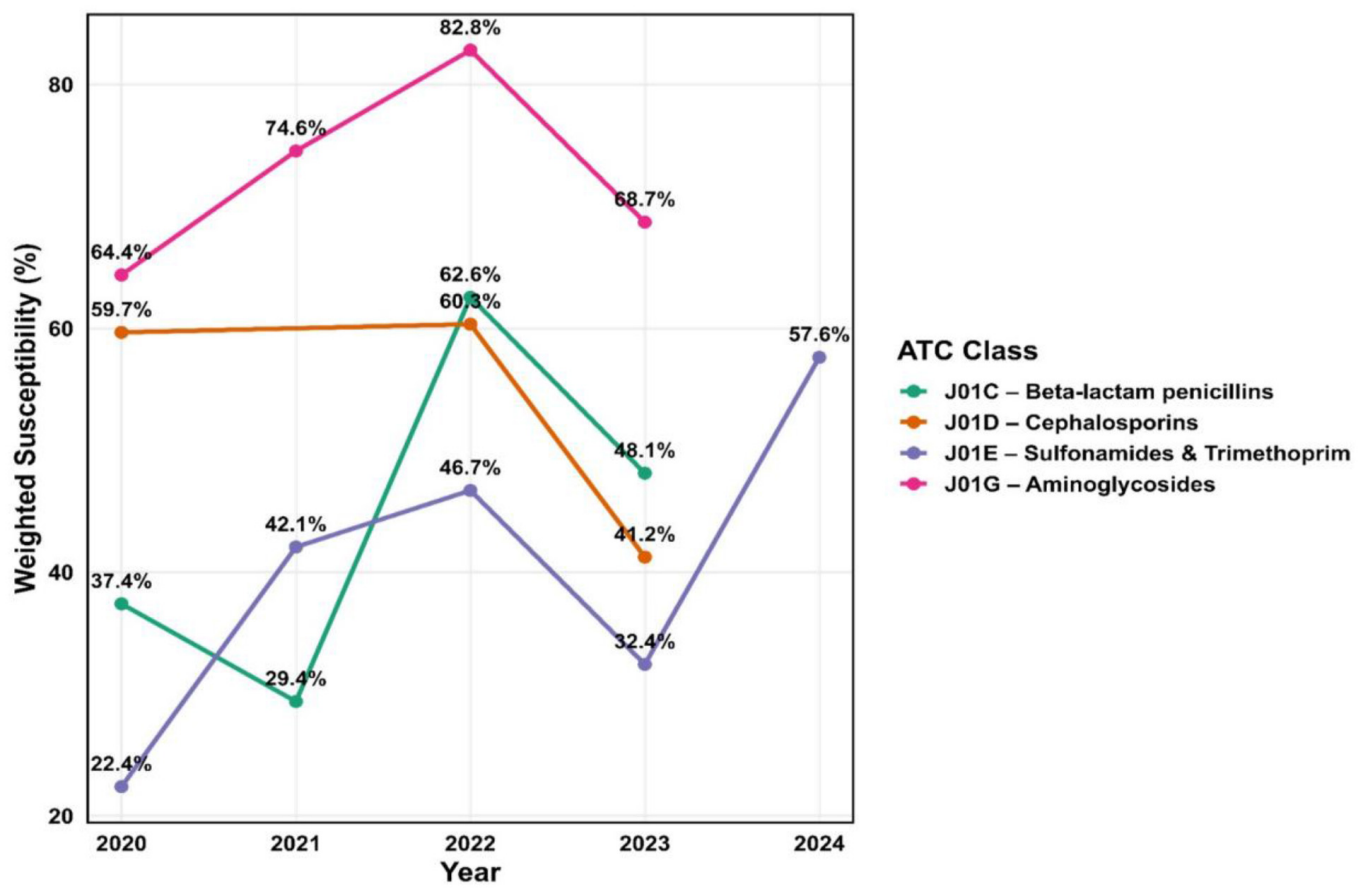
Year-wise weighted susceptibility trends of major WHO ATC antibiotic classes (J01C, J01D, J01E, J01G) from 2020 to 2024.

Of antibiotic classes analyzed between 2020 and 2024, aminoglycosides (J01G) consistently demonstrated the highest weighted susceptibility, ranging from 68.7 to 82.8%, with peak activity observed in 2022 (Figure 5). Beta-lactam penicillins (J01C) showed more variable performance, with susceptibility as low as 29.4% in 2021, an improvement in 2022, and a subsequent decline in 2023. Sulfonamides and trimethoprim (J01E) displayed a gradual increase, rising from 22.4 in 2020 to 57.6% in 2024, although the latter estimate was based on limited isolate numbers and should be interpreted with caution. Cephalosporins (J01D) showed relatively strong activity initially but declined notably by 2023.

## Discussion

This study offers a consolidated five-year overview of antimicrobial susceptibility patterns in Pakistan, integrating data across diverse clinical pathogens and antibiotic classes. By positioning these findings within the WHO AWaRe framework and the UNGA benchmark of ≥70% consumption from Access agents, we provide an evidence-based perspective on the feasibility of achieving this global stewardship target in the Pakistani setting.

Our analysis revealed that most Access antibiotics, particularly aminopenicillins such as ampicillin and amoxicillin-clavulanate, along with co-trimoxazole, demonstrated low susceptibility against major Gram-negative pathogens, including *Escherichia coli* and *Klebsiella spp*. This aligns with earlier evidence from Pakistan showing declining susceptibility of Escherichia coli to commonly used agents such as amoxicillin-clavulanate and trimethoprim-sulfamethoxazole (108–110). Ehsan et al. observed 77% resistance against amoxicillin in uropathogenic *E. coli*, corroborating our findings (111). Certain Access agents, such as nitrofurantoin for urinary tract isolates and amoxicillin-clavulanate for respiratory pathogens, retained relatively high susceptibility exceeding 70%. However, their pathogen-specific activity restricts their role in broad-spectrum empirical therapy. These findings align with the 2024 WHO Bacterial Priority Pathogen List (BPPL), which identified *E. coli, K. pneumoniae*, and *S. aureus* as critical global threats, reinforcing the significance of our results within the broader AMR landscape (112). Infection-specific studies from Pakistan, Iran, and Türkiye reported high resistance (>30%) to first-line antibiotics in sepsis, burns, and wound infections. Gram-negative isolates from burn infections showed particularly high resistance to aminoglycosides and carbapenems, with colistin resistance among *Klebsiella pneumoniae* in Pakistan reaching 81% (113, 114).

For Gram-positive organisms, susceptibility trends were variable. *Staphylococcus aureus* showed an improvement in 2022 but declined sharply by 2024, reflecting both variations in isolate numbers and the ongoing burden of MRSA and multidrug-resistant strains in Pakistan (115). A recent systematic review and meta-analysis reported a high resistance rate of *MRSA* (61%) in Pakistan, comparable to our findings (113). Whereas, the susceptibility proportion for *Pseudomonas spp*. exhibited a marked decline before culminating at the lowest 20% rate in 2024. This rapid decline in susceptibility could be attributed to the high emergence of Carbapenem-resistant *Pseudomonas aeruginosa*, which is classified as a high-priority pathogen in the WHO Bacterial Priority Pathogens List (BPPL) 2024 (112).

Class-level analysis further highlighted these challenges. Aminoglycosides (J01G) showed the highest and most consistent activity, with weighted susceptibility ranging from 64.4 to 82.8%, occasionally surpassing the 70% benchmark. Yet, systematic reviews indicate that aminoglycoside-modifying enzymes (AMEs) and efflux pump overexpression, especially in *E. coli, K. pneumoniae*, and *P. aeruginosa*, drive cross-resistance with β-lactams and fluoroquinolones, often leading to treatment failures and prolonged hospital stays (116). In contrast, beta-lactam penicillins (J01C) and cephalosporins (J01D) are among the most heavily prescribed agents in Pakistan (117), demonstrating variable susceptibility trends in our study, mirroring global concerns about overuse-driven resistance. Such findings emphasize that achieving the UNGA's ≥70% Access antibiotic consumption target may be unrealistic in Pakistan, given the low susceptibility of aminopenicillins and co-trimoxazole observed in our study. WHO has highlighted the importance of combining regulatory measures with research on innovative solutions, such as rapid diagnostics and alternative therapies (118). Although not directly assessed here, these strategies could play a supportive role in addressing the declining effectiveness of Access antibiotics demonstrated in our analysis.

Beta-lactam penicillins showed a fluctuating pattern, with susceptibility of 37.4% in 2020, declining to 29.4% in 2021, before rising markedly to 62.6% in 2022, and then dropping again to 48.1% in 2023. Similarly, cephalosporins exhibited inconsistent activity, with a weighted susceptibility of 59.7% in 2020, increasing slightly to 60.3% in 2022, but subsequently decreasing to 41.2% in 2023. These findings highlight instability in resistance patterns for these widely used antibiotic classes. Comparable observations were also reported in a study from Saidu Teaching Hospital, Swat, which analyzed WHO priority pathogens and highlighted increasing resistance to cephalosporins in Pakistan (119). Moreover, recent studies from our research group accentuated higher procured volumes and subsequent misemploying broad-spectrum cephalosporins, particularly Ceftriaxone, in health facilities of Punjab as an obvious reason behind augmented microbial resistance against these agents (120, 121). Such convergence of evidence underscores the urgent need for optimized prescribing practices and strengthened antimicrobial stewardship interventions to address the rising threat of resistance in these critical antibiotic groups. Several important data and reporting gaps were identified in the included studies. Notably, there were no studies from the provinces of Gilgit Baltistan and Baluchistan, leading to significant geographic underrepresentation. Additionally, a few studies from Punjab, Khyber Pakhtunkhwa, and Swat districts failed to specify city-level details. In terms of reporting completeness, 29.72% failed to specify patient type, and 18.91% did not report gender. Furthermore, the sample type was missing in 4.05% of studies. These omissions reduce the comparability and generalizability of findings and underscore the need for standardized data reporting framework**s** in AMR surveillance. Despite these limitations, our findings reinforce the WHO's call for coordinated action across healthcare systems and policymakers. Strengthening surveillance and stewardship remains essential to preserve antibiotic effectiveness and safeguard public health (118).

The implications of these findings are clear: strengthening national AMR surveillance to capture pathogen- and class-specific resistance trends is critical, as is integrating real-time susceptibility data into treatment guidelines. Stewardship efforts must also prioritize optimization of Access antibiotics, coupled with system-level measures such as regulating over-the-counter antibiotic sales, expanding diagnostic-driven prescribing, and enhancing infection prevention and control. Together, these interventions are essential to preserve the remaining efficacy of Access antibiotics and progressively align with the 70% benchmark.

A limitation of this scoping review is that some antibiotic–pathogen combinations in the dataset were associated with low or missing isolate counts, which may limit the representativeness of the findings and reduce the generalizability of observed susceptibility trends. Furthermore, the quality and quantity of available data were suboptimal and largely restricted to a few cities, potentially biasing the results and limiting nationwide applicability. Despite these limitations, the study provides a comprehensive overview of country-level antibiotic susceptibility patterns across multiple bacterial pathogens over several years. The use of weighted susceptibility calculations standardizes results across varying isolate counts, enhancing comparability. The multi-year and multi-center design allows for trend analysis and identifies key areas for antimicrobial stewardship interventions. By combining pathogen-specific and class-level analyses, the study generates actionable insights to guide evidence-based prescribing and support rational antibiotic use in Pakistan's healthcare settings.

## Conclusion

In this scoping review, we analyzed country-level susceptibility patterns in Pakistan and found persistently low susceptibility to Access antibiotics, limiting their reliability as first-line therapies against major bacterial pathogens. The findings indicate a clear deviation from the WHO's 70% Access utilization target, highlighting gaps between policy and practice (Box 1). Although the AWaRe framework serves as a global tool to guide antimicrobial stewardship, the observed susceptibility trends underscore weak implementation and inadequate microbiological infrastructure. Strong measures are needed to strengthen ASPs within hospitals, standardize surveillance, and ensure evidence-based prescribing to curb AMR and preserve antibiotic efficacy for future use. Furthermore, there is a need for more data from additional cities, as this review revealed that most cities in Pakistan are not providing sufficient published data on antimicrobial susceptibility.

Box 1 **Antibiotic susceptibility summary.**

- **High Susceptibility (>70%)** Amikacin, Nitrofurantoin, Chloramphenicol, Amoxicillin–clavulanate (for respiratory pathogens), Ampicillin (for respiratory pathogens), Doxycycline (*Klebsiella spp, Pseudomonas spp*).
- **Moderate Susceptibility (40–70%)** Cefazolin, Cephalexin, Amoxicillin (non-respiratory pathogens), Gentamicin (*Klebsiella spp*), Amikacin (*Klebsiella spp*), Doxycycline, Trimethoprim–sulfamethoxazole.
- **Low Susceptibility (< 40%)** Trimethoprim–sulfamethoxazole (most Gram-negatives), Trimethoprim, Amoxicillin–clavulanate (Gram-negatives), Ampicillin (Gram-negatives), Amoxicillin (Gram-negatives).

## Data Availability

The original contributions presented in the study are included in the article/Supplementary material, further inquiries can be directed to the corresponding author.

## References

[1] Salam MA Al-Amin MY Salam MT Pawar JS Akhter N Rabaan AA . Antimicrobial resistance: a growing serious threat for global public health. Healthcare. (2023) 11:1946. doi: 10.3390/healthcare11131946 37444780 PMC10340576

[2] Batool M Keating C Javed S Nasir A Muddassar M Ijaz UZ . cross-sectional study of potential antimicrobial resistance and ecology in gastrointestinal and oral microbial communities of young normoweight Pakistani individuals. Microorganisms. (2023) 11:279. doi: 10.3390/microorganisms11020279 36838244 PMC9965051

[3] Urban-Chmiel R Marek A Stepień-Pyśniak D Wieczorek K Dec M Nowaczek A . Antibiotic resistance in bacteria—A review. Antibiotics. (2022) 11:1079. doi: 10.3390/antibiotics11081079 36009947 PMC9404765

[4] Founou RC Founou LL Essack SY. Clinical and economic impact of antibiotic resistance in developing countries: a systematic review and meta-analysis. PLoS ONE. (2017) 12:e0189621. doi: 10.1371/journal.pone.0189621 29267306 PMC5739407

[5] Tabish SA. Recent trends in emerging infectious diseases. Int J Health Sci. (2009) 3:V–VII. 21475529 PMC3068824

[6] Naghavi M Vollset SE Ikuta KS Swetschinski LR Gray AP Wool EE . Global burden of bacterial antimicrobial resistance 1990–2021: a systematic analysis with forecasts to 2050. Lancet. (2024) 404:1199–226. doi: 10.1016/S0140-6736(24)01867-1 39299261 PMC11718157

[7] Chinemerem Nwobodo D Ugwu MC Oliseloke Anie C Al-Ouqaili MT Chinedu Ikem J Victor Chigozie U . Antibiotic resistance: the challenges and some emerging strategies for tackling a global menace. J Clin Lab Anal. (2022) 36:e24655. doi: 10.1002/jcla.24655 35949048 PMC9459344

[8] Bajwa M Afzal S Sheikh SA Saleem Z. Drug Inspector as an antibiotic steward: challenges and recommendations to implement national action plan of Pakistan on antimicrobial resistance. Expert Rev Anti Infect Ther. (2024) 22:907–20. doi: 10.1080/14787210.2024.2368825 38872588

[9] Bilal H Khan MN Rehman T Hameed MF Yang X. Antibiotic resistance in Pakistan: a systematic review of past decade. BMC Infect Dis. (2021) 21:1–19. doi: 10.1186/s12879-021-05906-1 33676421 PMC7937258

[10] WHO. The Who Aware (Access, Watch, Reserve) Antibiotic Book. Available online at: https://www.who.int/publications/i/item/9789240062382 (Accessed October 3, 2025).

[11] Saleem Z Godman B Azhar F Kalungia AC Fadare J Opanga S . Progress on the national action plan of Pakistan on antimicrobial resistance (AMR): a narrative review and the implications. Expert Rev Anti Infect Ther. (2022) 20:71–93. doi: 10.1080/14787210.2021.1935238 34038294

[12] Gandra S Kotwani A. Need to improve availability of “access” group antibiotics and reduce the use of “watch” group antibiotics in India for optimum use of antibiotics to contain antimicrobial resistance. J Pharm Policy Pract. (2019) 12:1–4. doi: 10.1186/s40545-019-0182-1 31346472 PMC6636108

[13] Saleem Z Hassali MA Godman B Hashmi FK Saleem F. Antimicrobial prescribing and determinants of antimicrobial resistance: a qualitative study among physicians in Pakistan. Int J Clin Pharm. (2019) 41:1348–58. doi: 10.1007/s11096-019-00875-7 31273588

[14] Iqbal MS Khan MF Farooqui S Khan S-U-D Vohra S Rasheed S . Antibiotic utilization and resistance according to the WHO AWaRe classification in intensive care units after COVID-19 third wave in Pakistan: findings and implications. Medicina. (2025) 61:481. doi: 10.3390/medicina61030481 40142292 PMC11944239

[15] Sheikh S Saleem Z Afzal S Qamar MU Raza AA Haider Naqvi SZ . Identifying targets for antibiotic stewardship interventions in pediatric patients in Punjab, Pakistan: point prevalence surveys using AWaRe guidance. Front Pediatr. (2025) 12:1469766. doi: 10.3389/fped.2024.1469766 39867700 PMC11759272

[16] Nasir N Mehmood BK Awan S Sattar S Tajuddin S. Evaluation of hospital antibiotic prescription patterns using WHO AWaRe Classification in Karachi, Pakistan. J Glob Antimicrob Resist. (2024) 39:29. doi: 10.1016/j.jgar.2024.10.091

[17] Kotwani A Joshi J Lamkang AS. Over-the-counter sale of antibiotics in India: a qualitative study of providers' perspectives across two states. Antibiotics. (2021) 10:1123. doi: 10.3390/antibiotics10091123 34572705 PMC8472180

[18] Akande-Sholabi W Oyesiji E. Antimicrobial stewardship: knowledge, perceptions, and factors associated with antibiotics misuse among consumer's visiting the community pharmacies in a Nigeria Southwestern State. J Pharm Policy Pract. (2023) 16:120. doi: 10.1186/s40545-023-00629-x 37821920 PMC10566051

[19] Abdullah S Rahman Su Muhammad F Mohsin M. Association between antimicrobial consumption and resistance rate of *Escherichia coli* in hospital settings. J Appl Microbiol. (2023) 134:lxac003. doi: 10.1093/jambio/lxac003 36626753

[20] Aldarhami A. Identification of novel bacteriocin against *Staphylococcus* and *Bacillus* species. Int J Health Sci. (2023) 17:15. doi: 10.1016/S0140-6736(24)018 37692990 PMC10484066

[21] Iqbal Z Sheikh AS Basheer A Hafsa HT Ahmed M Sabri AN . Antibiotic drug resistance pattern of uropathogens in pediatric patients in Pakistani population. Antibiotics. (2023) 12:395. doi: 10.3390/antibiotics12020395 36830305 PMC9952681

[22] Tawfick MM Adulall AK El-Kholy AA El Manakhly AR. Mutation-based fluoroquinolone resistance in carbapenem-resistant *Acinetobacter baumannii* and *Escherichia coli* isolates causing catheter-related bloodstream infections. Int J Health Sci. (2023) 17:18. 37151743 PMC10155246

[23] Saleem Z AMR battle in Pakistan: from national action plans to local failures. Arch Public Health. (2025) 83:1–3. doi: 10.1186/s13690-025-01568-6 40200283 PMC11980256

[24] Torumkuney D Jamil B Nizamuddin S Van Hasselt J Pirzada U Manenzhe R. Country data on AMR in Pakistan in the context of community-acquired respiratory tract infections: links between antibiotic susceptibility, local and international antibiotic prescribing guidelines, access to medicine and clinical outcome. J Antimicrob Chemother. (2022) 77:i18–25. doi: 10.1093/jac/dkac213 36065729 PMC9445852

[25] Iskandar K Molinier L Hallit S Sartelli M Hardcastle TC Haque M . Surveillance of antimicrobial resistance in low-and middle-income countries: a scattered picture. ARIC. (2021) 10:63. doi: 10.1186/s13756-021-00931-w 33789754 PMC8011122

[26] WHO. Global Antimicrobial Resistance Surveillance System: Manual For Early Implementation. Available online at: https://iris.who.int/handle/10665/188783 (Accessed October 3, 2025).

[27] Nations U. Political Declaration of the High-level Meeting on Antimicrobial Resistance. Available online at: https://www.un.org/pga/wp-content/uploads/sites/108/2024/09/FINAL-Text-AMR-to-PGA.pdf (Accessed November 21, 2025).

[28] Ortiz-Prado E Izquierdo-Condoy JS Mora C Vasconez-Gonzalez J Fernandez-Naranjo R. Poor regulation, desperation, and misinformation, a countrywide analysis of self-medication and prescription patterns in Ecuador during the COVID-19 pandemic. RSAP. (2023) 19:1579–89. doi: 10.1016/j.sapharm.2023.08.011 37659922

[29] Bhatti JM Raza SA Alam AF Khan YN Mala A Batool I . Antibiotic choices among healthcare professionals for enterococcal bacteremia with patterns of resistance and risk factors of mortality, in settings of poor antibiotic stewardship program—a five-year retrospective cohort study. BMC Infect Dis. (2023) 23:514. doi: 10.1186/s12879-023-08498-0 37544982 PMC10405468

[30] World Health Organization (WHO). Available online at: https://www.who.int/tools/atc-ddd-toolkit/atc-classification (Accessed November 21, 2025).

[31] Peters MD Marnie C Tricco AC Pollock D Munn Z Alexander L . Updated methodological guidance for the conduct of scoping reviews. JBI Evid Synth. (2020) 18:2119–26. doi: 10.11124/JBIES-20-00167 33038124

[32] Minnesota Statewide Acute Care Antibiogram. Available online at: https://www.health.state.mn.us/diseases/antibioticresistance/ac21antibio.pdf (Accessed April 2, 2026).

[33] BMJ 2021; 372. Available online at: https://www.bmj.com/content/372/bmj.n71 (Accessed April 2, 2026).

[34] Yaseen M Rashid S Naqvi S. Urinary tract infection in pregnant females attending antenatal clinics among middle socioeconomic settings. Professional Med J. (2020) 27:1636–41. doi: 10.29309/TPMJ/2020.27.08.4357

[35] Sarwar A Butt MA Hafeez S Danish MZ. Rapid emergence of antibacterial resistance by bacterial isolates from patients of gynecological infections in Punjab, Pakistan. J. Infect. Public Health. (2020) 13:1972–80. doi: 10.1016/j.jiph.2020.06.011 32605779

[36] Shaikh M Hanif M Gul R Hussain W Hemandas H Memon A. Spectrum and antimicrobial susceptibility pattern of micro-organisms associated with neonatal sepsis in a hospital in Karachi, Pakistan. Cureus. (2020) 12. doi: 10.7759/cureus.10924 33194490 PMC7657439

[37] Muhammad A Khan SN Ali N Rehman MU Ali I. Prevalence and antibiotic susceptibility pattern of uropathogens in outpatients at a tertiary care hospital. New Microbes New Infect. (2020) 36:100716. doi: 10.1016/j.nmni.2020.100716 32637123 PMC7330609

[38] Jan H Riaz M Shah D Hussain F Khan MM. Causative organisms of surgical site infections and their antimicrobial susceptibility patterns in a general surgical ward in peshawar. JSP (2021) 37:9–13.

[39] Mahmood T Tariq MA. ud din Zafir MB, Chishti MK, Shafee M, Rasool A. Frequency of common pathogens isolated from open fractures of the extremities and their antimicrobial sensitivity pattern. JPOA. (2020) 32:197–201.

[40] Rizvi ZA Jamal AM Malik AH Zaidi SMJ Rahim NUA Arshad D. Exploring antimicrobial resistance in agents causing urinary tract infections at a tertiary care hospital in a developing country. Cureus. (2020) 12. doi: 10.7759/cureus.9735 32944453 PMC7489772

[41] Hussain M Khan MNA Rehman K Mirza IA Rehman M Riaz S. Bacterial spectrum and antimicrobial pattern of blood stream infections associated with non-tunneled double lumen catheter in hemodialysis patients. Pak Armed Forces Med J. (2021) 71:1161–6. doi: 10.51253/pafmj.v71i4.4438

[42] Ahmad I Hussain MS Akhtar MS. Microbial spectrum and antibiotic sensitivity in cirrhotic patients with spontaneous bacterial peritonitis. Pak J Med Health Sci. (2021) 15:3123–5. doi: 10.53350/pjmhs2115113123

[43] Ishtiaq S Ahmed I. Susceptibility pattern of bacterial isolates from surgical site infections in a tertiary care hospital at Rawalpindi. JIIMC. (2021) 16:224–31.

[44] Zeshan B Karobari MI Afzal N Siddiq A Basha S Basheer SN . The usage of antibiotics by COVID-19 patients with comorbidities: the risk of increased antimicrobial resistance. Antibiotics. (2021) 11:35. doi: 10.3390/antibiotics11010035 35052912 PMC8772884

[45] Fatima K Ahmad K Ejaz A Mir N Ch S. Outbreak of Pan-resistant Acinetobacter Species in Intensive Care Units of a tertiary care hospital. Proteus. (2022) 1:0.5. doi: 10.53350/pjmhs2216443

[46] Bhatti RA Jakhrani MR Shaikh A Shaikh AH Bhutto I Qureshi M. Frequency of various causative bacterial organisims and their culture sensitivity pattern in patients with open tibial fracture. PJMHS. (2022) 16:217–9. doi: 10.53350/pjmhs22164217

[47] Rana MA Siddiqui MH Ahmad A Qayyum MA Iqbal W Iqbal J . Multidrug resistant *E. coli* in patients with urinary tract infections presenting to internal medicine clinics of two tertiary care hospitals in Lahore, tip of the iceberg. PJMHS. (2022) 16:363. doi: 10.53350/pjmhs22162363

[48] Imdad Z Maqbool T Rehman A Naz I Ikram S. Cultural sensitivity of sputum bacteria involved in chronic lung disease and type of bacteria involved in chronic lung disease sputum. PJMHS. (2022) 16:702. doi: 10.53350/pjmhs221610702

[49] Idrees MM Rasool MF Imran I Khalid A Saeed A Ahmad T . A cross-sectional study to evaluate antimicrobial susceptibility of uropathogens from South Punjab, Pakistan. Infect Drug Resist. (2022) 15:1845–55. doi: 10.2147/IDR.S356489 35450113 PMC9017698

[50] Anwar Y Ullah F Yasin M Basit A Ullah I Shah SF . Increasing antibiotic resistant pattern in clinical bacterial isolates, from tertiary care hospital, Hayatabad medical complex, Peshawar, Pakistan: increasing antibiotics resistance in Hayat Abad medical complex. Pak biomed j. (2022) 5:91–5. doi: 10.54393/pbmj.v5i1.177

[51] Anwar I Rafiq S Ahmad K Mubeen MU Mudassar A Kamran M. Antimicrobial susceptibility pattern of *E. coli* in patients with urinary tract infection at a tertiary care hospital, Rawalpindi. PJMHS. (2022) 16:967. doi: 10.53350/pjmhs22166967

[52] Ali A Rahman N Adeeb H Ullah I. Frequency and antimicrobial resistance profile of salmonella typhi isolated from district buner. J Med Sci. (2022) 30:185–9. doi: 10.52764/jms.22.30.3.5

[53] Hussain MA Akram S Khan MA Nawaz S Ali S Amir M. Neonatal sepsis; incidence and microbiological profile along with antibiotic sensitivity of causative microorganisms. Life Sci. (2023) 4:6. doi: 10.37185/248

[54] Karmani JK Ahmad A Iqbal M Arshad N. Antimicrobial sensitivity pattern of urine culture isolate in a tertiary care hospital. Pak J Med Health Sci. (2023) 17:61–6. doi: 10.53350/pjmhs202317261

[55] Jamal MM Arif A Sarfaraz A Jamal J Mahmood S Musarrat M. Uropathogens and their culture sensitivity pattern in children with urinary tract infection. Pak Armed Forces Med J. (2023) 73:1137. doi: 10.51253/pafmj.v73i4.9005

[56] Sherazi F Naz I Afridi RU Ambareen A Iqbal F Iqbal S. Multiple drug resistance, extensively drug resistance typhoid fever and disease spectrum in pediatric population presenting with fever without localizing signs (FWLS): a cross sectional study. KJMS. (2023) 16:103–8. doi: 10.70520/kjms.v16i2.460

[57] Akhtar A Fatima N Anwar I Shahbaz A Durrani T Fatima S . Evaluation of antibiotic resistance in pediatric patients suffering from cancer. J Clin Immunol Microbiol. (2023) 4:1–10. doi: 10.46889/JCIM.2023.4203

[58] Hussain A Rub Abidi SA Us Saqlain HA Lakhani S Ahmed Majoka MW Keerio NH. Common pathogen frequency and antimicrobial sensitivity pattern in open fractures of the extremities. J Pharm Negat Results. (2023) 14.

[59] Iqbal F Nasreen F Iqbal S Naz I Elahi A Rehman F. Bactericidal effect of antibiotics against bacteria causing urinary tract infection among children. PJMHS. (2023) 17:824. doi: 10.53350/pjmhs2023171824

[60] Zeb S Bakhtiar S Mehmood Z Zeb L Awan MB Chamkani ZU. Pattern of antibiotic resistance in stroke patients with UTI and its determinants. J Popul Ther Clin Pharmacol. (2023) 30:989–94. doi: 10.54112/bcsrj.v2023i1.470

[61] Altaf U Saleem Z Akhtar MF Altowayan WM Alqasoumi AA Alshammari MS . Using culture sensitivity reports to optimize antimicrobial therapy: findings and implications of antimicrobial stewardship activity in a hospital in Pakistan. Medicina. (2023) 59:1237. doi: 10.3390/medicina59071237 37512049 PMC10384799

[62] Bashir N Dablool AS Khan MI Almalki MG Ahmed A Mir MA . Antibiotics resistance as a major public health concern: a pharmaco-epidemiological study to evaluate prevalence and antibiotics susceptibility-resistance pattern of bacterial isolates from multiple teaching hospitals. J Infect Public Health. (2023) 16:61–8. doi: 10.1016/j.jiph.2023.09.019 37880004

[63] Ali R. Antibiotic susceptibility patterns of bacteria isolated from patients with community acquired urinary tract infections. Professional Med J. (2023) 30:1501–5. doi: 10.29309/TPMJ/2023.30.11.7883

[64] Abbas G Khan HA Iqbal S Nabi A. Bacterial isolates and their sensitivity patterns in patients with diabetic foot ulcers. J Med Sci. (2023) 31:4–9. doi: 10.52764/jms.23.31.1.1

[65] Waheed A Afridi I Tareen A Khan M Khan D. Bacteria causing community acquired superficial skin infections in tertiary care hospitals and their antibiotics susceptibility patterns. JPAD (2023) 33:108–15. Available online at: https://www.jpad.com.pk/index.php/jpad/article/view/2204 (Accessed January 23, 2024).

[66] Gul S Mir N Fatima K Tahir S Younis N Khatoon F. Catheter-related infections in Hemodialysis: Frequency and microbiological profile patients undergoing antimicrobial lock therapy with gentamicin for prophylaxis. Biol Clin Sci Res J. (2023) 2023:247. doi: 10.54112/bcsrj.v2023i1.247

[67] Atif M. Isolation and identification of bacterial pathogens associated with indwelling devices and their antimicrobial susceptibility. TRS. (2023):1177–89.

[68] Saleem Z Haseeb A Abuhussain SSA Moore CE Kamran SH Qamar MU . Antibiotic susceptibility surveillance in the Punjab province of Pakistan: findings and implications. Medicina. (2023) 59:1215. doi: 10.3390/medicina59071215 37512028 PMC10383515

[69] Afzal T Khalid T Afzal N Inam F Bajwa B Goraya SI . Antibiotic sensitivity pattern of deep skin and soft tissue infections in Pakistan. Professional Med J. (2024) 31:1368–74. doi: 10.29309/TPMJ/2024.31.09.8148

[70] Aman BS Taj MK Taj I Azam S Khan S Rashid R. The bacterial profile and antibiotic susceptibility in skin and soft tissue infections at a tertiary care hospital of Quetta, Pakistan. JPMA. (2024) 74:1249–53. doi: 10.47391/JPMA.10261 39028049

[71] Khalid N Akbar Z Mustafa N Akbar J Saeed S Saleem Z. Trends in antimicrobial susceptibility patterns of bacterial isolates in Lahore, Pakistan. Front Antibiot. (2023) 2:1149408. doi: 10.3389/frabi.2023.1149408 39816653 PMC11731995

[72] Akhtar ST Naqvi IH Khan O Hasmi STA Rizvi SNZ. Heading towards threat of resistant super bug…. current pattern of antimicrobial sensitivity to salmonella typhi in Karachi. Professional Med J. (2020) 27:1070–3. doi: 10.29309/TPMJ/2020.27.05.4389

[73] Jahanzeb M Nismat J Farhan M Uzma I Zubair A. Microbial resistance in urinary tract infections. Cureus. (2020) 12. 32542163 10.7759/cureus.8110 PMC7292691

[74] Khurshid M Rasool MH Ashfaq UA Aslam B Waseem M Xu Q . Dissemination of blaOXA-23-harbouring carbapenem-resistant *Acinetobacter baumannii* clones in Pakistan. J Glob Antimicrob Resist. (2020) 21:357–62. doi: 10.1016/j.jgar.2020.01.001 32006748

[75] Khurshid M Rashid A Husnain M Rasool MH Waqas U Saeed M . *In-vitro* assessment of the therapeutic potential of polymyxins and tigecycline against multidrug-resistant acinetobacter isolates from infected wounds. JAMC. (2020) 32:459–64. 33225644

[76] Khan MI Xu S Ali MM Ali R Kazmi A Akhtar N . Assessment of multidrug resistance in bacterial isolates from urinary tract-infected patients. J Radiat Res Appl Sci. (2020) 13:267–75. doi: 10.1080/16878507.2020.1730579

[77] Durrani A. Emergence of multi drug resistant *Salmonella typhi* as epidemic among lower Sindh regions patients of Pakistan. LMRJ. (2020) 2:8–94. doi: 10.38106/LMRJ.2020.2.4.04

[78] Maka G Shah S Bano S Tunio SA. Antibiotic susceptibility profiling of Gram-negative bacteria causing upper respiratory tract infections in Hyderabad, Sindh. JLBSR. (2020) 1:12–5. doi: 10.38094/jlbsr112

[79] Zahra N Zeshan B Qadri MMA Ishaq M Afzal M Ahmed N. Phenotypic and genotypic evaluation of antibiotic resistance of *Acinetobacter baumannii* bacteria isolated from surgical intensive care unit patients in Pakistan. Jundishapur J Microbiol. (2021) 14:e113008. doi: 10.5812/jjm.113008

[80] Ullah R Amir M Anjum S Rehman MU Hasan TN Naqvi SS . Presence of T3SS (exoS, exoT, exoU and exoY), susceptibility pattern and MIC of MDR-Pseudomonas aeruginosa from burn wounds. J Infect Dev Ctries. (2023) 17:1130–7. doi: 10.3855/jidc.17580 37699096

[81] Shams H Suliman M Noor U Aziz A Khanum S Naz N . Extended spectrum B-Lactamase (ESBL) and metallo B-lactamase (MBL) production in gram-negative bacteria isolated from urinary tract infection patients. JSMC. (2023) 13:46–53. doi: 10.52206/jsmc.2023.13.2.738

[82] Ahmad J Ahmad S. Simultaneous application of non-antibiotics with antibiotics for enhanced activity against multidrug resistant *Pseudomonas aeruginosa*. J. Pharm. Negat. Results (2023) 14. doi: 10.47750/pnr.2023.14.03.506

[83] Haseeb A Saleem Z Altaf U Batool N Godman B Ahsan U . Impact of positive culture reports of *E. coli* or *MSSA* on de-escalation of antibiotic use in a teaching hospital in Pakistan and the implications. Infect Drug Resist. (2023) 16:77–86. doi: 10.2147/IDR.S391295 36636371 PMC9831081

[84] Khan NB Shehzadi M Hameed H Nazeer MF Amjad U Rizwan M . study on *Escherichia coli* Isolated from urogenital tract infections, emphasizing their occurrence and antibiograms. PJMHS. (2023) 17:323. doi: 10.53350/pjmhs2023173323

[85] Ahmad A Afridi IG. Antimicrobial sensitivity of salmonella typhi in children-a single center study. Professional Med J. (2023) 30:1442–4. doi: 10.29309/TPMJ/2023.30.11.7465

[86] Khan HA Umam S Yousaf M Rashid A Abbas G Shah BM . Frequency of *E. coli* and its sensitivity to nitrofurantion in patients with urinary tract infection. J Med Sci. (2021) 29. doi: 10.52764/jms.21.29.2.13

[87] Kamran M Choudary MA Amin H Asghar S Shahid A Zafar S . Antimicrobial susceptibility pattern of pseudomonas aeruginosa isolated from clinical and environmental sources in Punjab, Pakistan: antimicrobial susceptibility pattern of *Pseudomonas aeruginosa*. Pak biomed j. (2022) 5:34–8. doi: 10.54393/pbmj.v5i3.349

[88] Raza A Munir S Rashid N Muzafar R Khan S. Prevalence and antimicrobial sensitivity pattern of gram negative rods in blood cultures: a tertiary care hospital study. PJMHS. (2022) 16:124. doi: 10.53350/pjmhs22166124

[89] Noor U Suliman M Shams H Sultan A Khan SH. Prevalence and phenotypic detection of carbapenem and multi drug resistant of *E. coli* in urinary tract infection patients in district swat: detection of carbapenem and multi drug resistant of *E. coli*. PJHS. (2022) 3:243–7. doi: 10.54393/pjhs.v3i06.367

[90] Hafeez SB Ahmed A Akhtar A Ishtiaq W Javed NUS Abbas K . Catheter-related bloodstream infection with femoral central access versus internal jugular access in patients admitting to medical intensive care unit. Cureus. (2022) 14. doi: 10.7759/cureus.29416 36304372 PMC9586494

[91] Aqib B Bilal R. Prevalence of multidrug-resistance of *Escherichia coli* in urinary tract infection patients visiting Rehman Medical Hospital, Peshawar, Pakistan. Int. J. Life Sci. (2022). Available online at: https://ijlsci.in/ls/index.php/home/article/view/609 (Accessed January 24, 2024).

[92] Afzal E Khan S Iqbal MK Ahmad T. Prevalence of urinary tract infection in children with cerebral palsy: experience at tertiary care centre. Professional Med J. (2022) 29:1866–71. doi: 10.29309/TPMJ/2022.29.12.6925

[93] Khan MR Azam S Ahmad S Ali Q Liaqat Z Rehman N . Molecular Characterization and epidemiology of antibiotic resistance genes of β-lactamase producing bacterial pathogens causing septicemia from tertiary care hospitals. Antibiotics. (2023) 12:617. doi: 10.3390/antibiotics12030617 36978484 PMC10045492

[94] Taj Z Rasool MH Khurshid M Aslam B Qamar MU. Insights into the intersection of biocide resistance, efflux pumps, and sequence types in carbapenem-resistant *Acinetobacter baumannii*: a multicenter study. Pathogens. (2023) 12:899. doi: 10.3390/pathogens12070899 37513746 PMC10383717

[95] Taj S Yasmin S Mubariz R Butt T Bari A Musarat U. Antibacterial susceptibility pattern of gram-negative ESKAPE pathogens isolated from hospitalized patients. JIIMC. (2020) 15:231–5.

[96] Fida S Mansoor H Saif S Iqbal J Khan AQ. Clinical perspectives of multiple and extensively drug-resistant typhoid; result from a tertiary care hospital from Pakistan. J Infect Dev Ctries. (2021) 15:530–7. doi: 10.3855/jidc.13539 33956653

[97] Ejaz H Ahmad M Younas S Junaid K Abosalif KOA Abdalla AE . Molecular epidemiology of extensively-drug resistant *Acinetobacter baumannii* sequence type 2 co-harboring bla NDM and bla OXA from clinical origin. Infect Drug Resist. (2021) 14:1931–9. doi: 10.2147/IDR.S310478 34079303 PMC8164867

[98] Ejaz H Qamar MU Junaid K Younas S Taj Z Bukhari SNA . The molecular detection of class b and class d carbapenemases in clinical strains of *Acinetobacter calcoaceticus-baumannii* complex: the high burden of antibiotic resistance and the co-existence of carbapenemase genes. Antibiotics. (2022) 11:1168. doi: 10.3390/antibiotics11091168 36139948 PMC9494970

[99] Kamran M Iqbal U Saleem M Khan AA Haq MU Ahmad M . Antibiogram and prevalence Of ESBL genes in *Escherichia coli* from clinical specimen. Blood. (2022) 23:19.0.

[100] Saeed M Rasheed F Rasool MH Hayat S Khurshid M. Carbapenem-resistant *Burkholderia cepacia* complex isolates carrying blaNDM– 1 and blaNDM– 5 in ventilator-associated pneumonia patients and contaminated ventilator tubing. Transbound Emerg Dis. (2024) 2024:3352135. doi: 10.1155/2024/3352135 40303040 PMC12016991

[101] Ullah A Zeb MA Saidal A Malik A Ahmad T. Methicillin resistant *Staphylococcus aureus*: antibiotic resistant trend in Hayatabad medical complex Peshawar. Ann Allied Health Sci. (2020) 6:26–31.

[102] Khan M Komal W Saleem MA Ahmad N Rafaque Z Kabir S . Colonization and antibiotic resistance profiling of methicillin resistant *Staphylococcus aureus* (Mrsa) in Patients from tertiary. J Microbiol Mol Genet. (2020) 1:31–9. doi: 10.52700/jmmg.v1i3.11

[103] Hashmani S Patoli BB Kumari N Patoli AA Jabeen S. The resistance pattern of *Staphylococci* against beta-lactam group of antibiotics in Hyderabad, Pakistan. RMJ. (2021) 46:18–21.

[104] Abbas A Sarwar S. Antibiotic susceptibility and resistance of clinical isolates against various antibiotics: antibiotic susceptibility and resistance. MARKHOR. (2022) 29–32. doi: 10.54393/mjz.v3i02.57

[105] Mahmood I Shahid M Hanif F Manzoor M Awais M Adnan M. Frequency and antibiotic susceptibility of bacteria isolated in patients with blepharitis. Pak Armed Forces Med J. (2023) 73:S338. doi: 10.51253/pafmj.v73iSUPPL-2.8209

[106] Khalid F Iqbal MD Tariq TM Saeed N Sadaf S Akhtar A. Antibiogram of *Staphylococcus aureus* among clinical isolates at a tertiary care hospital in Lahore. Pak Postgrad Med J. (2023) 34:135–8. doi: 10.51642/ppmj.v34i03.607

[107] Methodology WCCfDS. ATC/DDD Index 2025. Available online at: https://atcddd.fhi.no/atc_ddd_index/ (Accessed November 22, 2025).

[108] Bangash K Mumtaz H Mehmood M Hingoro MA Khan ZZ Sohail A . Twelve-year trend of *Escherichia coli* antibiotic resistance in the Islamabad population. Ann Med Surg. (2022) 78:103855. doi: 10.1016/j.amsu.2022.103855 35734722 PMC9207067

[109] Khatoon I Khanam S Azam A Qadeer S Naz S Hassan NU. Incidence pattern, antibiotic susceptibility pattern and associated risk factors of bacterial uropathogens among general population of Pakistan. Infect Drug Resist. 2023:4995–5005. doi: 10.2147/IDR.S418045 37551281 PMC10404436

[110] Afzaal M Haroon M Afridi MF Haseeb A. ul Hassan F, Zahid M, et al. 18 detection of extended spectrum beta lactamase from multidrug resistance *Escherichia coli* from various clinical sample in district Peshawar, Pakistan. Pure appl biol. (2020) 9:2383–90. doi: 10.19045/bspab.2020.90252

[111] Ehsan B Haque A Qasim M Ali A Sarwar Y. High prevalence of extensively drug resistant and extended spectrum beta lactamases (ESBLs) producing uropathogenic *Escherichia coli* isolated from Faisalabad, Pakistan. World J Microbiol Biotechnol. (2023) 39:132. doi: 10.1007/s11274-023-03565-9 36959469 PMC10036249

[112] Sati H Carrara E Savoldi A Hansen P Garlasco J Campagnaro E . The WHO Bacterial Priority Pathogens List 2024: a prioritisation study to guide research, development, and public health strategies against antimicrobial resistance. Lancet Infect Dis. (2025). doi: 10.1016/S1473-3099(25)00118-5 40245910 PMC12367593

[113] Mathu R Diago-Navarro E Lynch E Degail M-A Ousley J Kanapathipillai R . Antibiotic resistance in the Middle East and Southern Asia: a systematic review and meta-analysis. JAC-AMR. (2025) 7:dlaf010. doi: 10.1093/jacamr/dlaf010 39973906 PMC11836886

[114] Khan M Khattak MT Gul A Riaz M. tu Zahra F. A comparable risk of extensively drug-resistant typhoid fever in the pediatric cohort during the COVID-19 pandemic. Int J Health Sci. (2024) 18:24. PMC10768466 38188899

[115] Khatoon A Hussain SF Shahid SM Sidhwani SK Khan SA Shaikh OA . Emerging novel sequence types of *Staphylococcus aureus* in Pakistan. J Infect Public Health. (2024) 17:51–9. doi: 10.1016/j.jiph.2023.10.036 37992434

[116] Zeb U Naz F Ahmed M Ishfaq S Sheryar M Arsalan M. Multi-pathogen aminoglycoside resistance: a dual approach in investigating bacteria across various clinical infections. PJMLS. (2024) 7:537–44. doi: 10.31580/pjmls.v7i3.3147

[117] Abdullah S Saleem Z Godman B Hashmi FK Haseeb A Al-Rawi MBA . Surge of branded generics and antimicrobial resistance: analyzing the antibiotic market dynamics in Pakistan through the WHO essential medicines and AWaRe lens. Expert Rev. Anti. Infect. Ther. (2025) 23:513–21. doi: 10.1080/14787210.2025.2511958 40418538

[118] Krishnaprasad VH Nayak V Kumar S. World health organisation's bacterial pathogen priority list (BPPL) 2017 and BPPL 2024 to combat global antimicrobial resistance crisis: ‘challenges and opportunities'. J Antimicrob Chemother. 2025:dkaf167. doi: 10.1093/jac/dkaf167 40424180

[119] Ali I Mufti S Taj N Ashfaq M Sharif MJH Khan Q . The growing threat of carbapenem and cephalosporin resistance: antimicrobial resistance patterns of who priority pathogens in a tertiary care setting. JAMC. (2025) 37:91–6. doi: 10.55519/JAMC-01-14380

[120] Afzal S Bajwa M Ahmed N Jabeen J Haroon MS Mushtaq RMZ . The antibiotic procurement saga: a long-neglected stewardship target to combat antimicrobial resistance in Pakistan. AMR. (2025) 14:7. doi: 10.1186/s13756-025-01521-w 39920829 PMC11806573

[121] Afzal S Bajwa M Haroon MS Arooj H Mushtaq RMZ Saleem Z. Unequal budgeting and limited WHO AWaRe Access antibiotics in Punjab: procurement policy gaps in Pakistan's National Action Plan against antimicrobial resistance. Arch Public Health. (2025) 83:1–17. doi: 10.1186/s13690-025-01720-2 41131624 PMC12548248

