# Supplementary material for: Can Pakistan achieve the UNGA 70% antibiotic access target? A scoping review of susceptibility data
Source: Front Public Health. 2026 May 1;14:1778876. doi: 10.3389/fpubh.2026.1778876 (PMC13175973; doi:10.3389/fpubh.2026.1778876)
Supplement: Supplementary file 1 [file Data_Sheet_1.docx]

**Can Pakistan Achieve the UNGA 70% Antibiotic Access Target? A Scoping Review of Susceptibility Data**

**Supplementary Table S1.** The number of studies classified as patient type, gender, age groups of gram-positive and gram-negative isolates, and the type of sample taken included in the present study

**Supplementary Table S2.** Main findings and characteristics of included studies (2020-2024)

**Supplementary Table S3.** Common microorganisms and Access group antibiotics in included studies

**Supplementary Table S1:** The number of studies classified as patient type, gender, age groups of gram-positive and gram-negative isolates, and the type of sample taken included in the present study.

| **Characteristics** | **No of studies** | **References** |
| --- | --- | --- |
| **Sample type** | | |
| Body fluids | 30 (40.54%) | [1-30] |
| Samples taken with a swab | 13 (17.56%) | [31-43] |
| Surgical devices | 2 (2.70%) | [44, 45] |
| Multiple sample types | 26 (35.13%) | [46-71] |
| Not mentioned | 3 (4.05%) | [72-74] |
| **Patient type** | | |
| Inpatients | 28 (37.83%) | [2, 5, 11, 16, 18, 33, 41, 44-48, 50-52, 55-58, 60-63, 65, 70, 72-74] |
| Outpatients | 16 (21.62%) | [1, 3, 4, 6-8, 14, 15, 17, 19, 21, 22, 30-32, 42] |
| Both | 8 (10.81%) | [9, 10, 20, 27, 36, 39, 49, 59] |
| Not mentioned | 22 (29.72%) | [12, 13, 23-26, 28, 29, 34, 35, 37, 38, 40, 43, 53, 54, 64, 66-69, 71] |
| **Gender** | | |
| Female | 2 (2.70%) | [1, 31] |
| Male | 1 (1.35%) | [44] |
| Both | 57 (77.02%) | [2-24, 26-30, 32-37, 39-41, 43, 45-51, 56, 57, 60, 62, 66-69, 71-74] |
| Not mentioned | 14 (18.91%) | [25, 38, 42, 52-55, 58, 59, 61, 63-65, 70] |
| **Age group** | | |
| Adults | 13 (17.56%) | [1, 5, 12, 16, 17, 22, 32, 34-36, 44, 46, 58] |
| Pediatric | 8 (10.81%) | [4, 7, 15, 21, 24, 28, 50, 72] |
| Pediatric+ Adults | 16 (21.62%) | [3, 6, 10, 18, 20, 23, 31, 33, 40, 43, 47, 49, 51, 56, 60, 74] |
| Neonates | 2 (2.70%) | [2, 11] |
| Neonates+ Pediatric | 2 (2.70%) | [13, 14] |
| No age limit/ All age groups | 11 (14.86%) | [8, 27, 29, 37, 39, 41, 65, 66, 68, 69, 73] |
| Not mentioned | 22 (29.72%) | [9, 19, 25, 26, 30, 38, 42, 45, 48, 52-55, 57, 59, 61-64, 67, 70, 71] |

**Supplementary Table S2:** Main findings and characteristics of included studies (2020-2024)

| **Author Name /Year**  **/Reference** | **Location** | **Healthcare setting/ Department** | **Disease/ Patient type** | **Isolated microbes** | **Study Duration** | **Sample Size** | **Sample Site** | **Findings** | **Conclusion** |
| --- | --- | --- | --- | --- | --- | --- | --- | --- | --- |
| Samina Fida et al.2020  [30] | Lahore | OPD/ Division of Medicine, Combined Military Hospital (CMH) | Patients with culture positive S. typhi and Paratyphi infection | Salmonella Typhi=47 (90.4%)  Salmonella Paratyphi=5 (9.6%) | April 2019 to October 2019 | 52 | Blood | Males=40 (76.9%)  Females=12 (23.1%)  Non-resistant=4 (7.7%)  MDR=11 (21.2%)  XDR=37 (71.2%) | The need for efficient infection control and prudent antibiotic usage is underscored by the growing resistance and difficulties of typhoid fever, especially in developing nations like Pakistan. |
| Shazia Taj et al. 2020  [61] | Rawalpindi | Pakistan Railway hospital (PRH)/ Microbiology lab | Hospitalized patients | Gram-negative ESKAPE pathogens=190 (59.3%) | 1^st^ September 2018 to 1^st^ September 2019 | 320 | Urine, blood, pus, wound, throat infection, nose infection, effusions, CSF and sputum | Enterobacteriaceae= 58 (30.5%)  Non- Enterobacteriaceae= 132 (69.4%)  Acinetobacter baumannii=68 (36%)  P. aeruginosa=64 (33.7%)  K. pneumonia=50 (26.3%)  Enterobacter spp=8 (4.2%) | The VITEK-2 compact system aids in identifying and testing Gram-negative ESKAPE pathogens quickly, reducing antibiotic consumption, with Enterobacter spp. |
| Mahjabeen Yaseen et al. 2020  [1] | Karachi | Jinnah Medical College Hospital/ Antenatal clinic | UTI among pregnant women | E. coli=26 (54.2%)  K. pneumoniae=8 (16.7%)  Pseudomonas=6 (12.5%)  Enterococcus=4 (8.3%)  S. aureus=4 (8.3%) | January 2017 to December 2017 | 564 | Urine | Culture positive=48 (8.5%)  Overall sensitivity:  Nitrofurantoin=42 (87.5%)  Amoxicillin clavulanic acid=28 (58.3%)  Ampicillin=15 (31.3%) | Nitrofurantoin is highly effective and have high sensitivity. Amoxicillin/clavulanic acid also yields good results. |
| Mehmood Shaikh et al. 2020  [2] | Karachi | Kharadar General Hospital/ Neonatology unit | Neonatal Sepsis | S. aureus=81 (50.4%)  Enterococci=2 (1%)  Pseudomonas=42 (25.7%)  Klebsiella=17 (10.5%)  E. coli=11 (6.7%)  Acinetobacter=9 (5.7%) | 1st January 2017 to 30th June 2019 | 1960 | Blood | Positive blood culture=162 (8.26%)  Gram-positive bacteria=83 (51.5%)  Gram-negative bacteria=79 (48.5%) | Because microorganisms and patterns of antibiotic sensitivity evolve, more local data and institutional-based guidelines are needed for neonatal sepsis. |
| Ammar Sarwar et al. 2020  [31] | Lahore | Two private and two government hospital laboratories/ Gynecological clinics | Gynecological infections | E. coli=154 (41.6%)  S. aureus=57 (15.4%)  CoNS=45 (12.2%)  Streptococcus=29 (7.8%)  Pseudomonas=29 (7.8%)  Klebsiella=17 (4.6%)  P. aeruginosa=16 (4.3%)  S. epidermidis=13 (3.5%)  Acinetobacter=10 (2.7%) | January 2011 to June 2017 | 520 | HVS from female genitalia | Culture positive=370 (71.15%)  Gram-positive bacteria=144 (38.9%)  Gram-negative bacteria=226 (61.1%)  E. coli, S. aureus, CoNS, Streptococcus, Pseudomonas=92.7% of isolates | Antibiotic resistance in Pakistan is increasing indicating a scarcity of chemotherapeutic agents for life-threatening infections, necessitating antibiotic rationalization and culture-sensitivity testing. |
| Syed Tahseen Akhtar et al. 2020  [18] | Karachi | Mamji Hospital/ Department of medicine department | Typhoid | Salmonella typhi=72 (100%) | Jan 2019 till July 2019 | 72 | Blood | Less than 18 years=30 (42%)  18 to 40 years=37 (51%)  Above 40 years=5 (7%)  Majority in males=43 (60%) | Most of patients suffered from prolonged drug-resistant typhoid, and some even had multidrug resistance. Quinolones and others showed high resistance. |
| A. Muhammad/et al. 2020  [3] | Peshawar | Khyber Teaching Hospital/ Department of Medical Laboratory technology | Patients having Symptomatic UTI | E. coli=198 (68.3%)  K. pneumoniae=27 (9.3%)  Proteus=6 (2.1%)  P. aeruginosa=12 (4.1%)  S. aureus=14 (4.8%)  MRSA=16 (5.5%)  Staphylococcus=9 (3.1%)  Enterococcus=8 (2.8%) | January to July 2019 | 804 | Midstream Urine | Culture positive urinary pathogens=290 (36.1%)  Culture negative=514 (63.9%)  E. coli ESBL=173 (87.4%)  K. pneumoniae ESBL=23 (85.2%)  Pseudomonas ESBL=6 (50%)  Proteus ESBL=5 (83.3%) | UTIs, primarily in women, are a major public health issue with E. coli, K. pneumoniae, and S. aureus being the most prevalent antibiotic-resistant organisms. |
| Aman Ullah et al. 2020  [71] | Peshawar KPK | Hayatabad Medical Complex/ NM | Community acquired infection | MRSA | October 2015 to October 2017 | 200 | Blood, ear swab, HVS, pus, tissue, urine, wound swabs | MRSA percentage in:  Pus=164 (82%)  Blood=22 (11%)  Urine=6 (3%)  Wound swabs=3 (1.5%)  HVS=2 (1%)  Tissue=2 (1%)  Ear Swab=1 (0.5%) | MRSA requires control through awareness, antimicrobial policies, prescribed antibiotic use, proper diagnosis, preventive guidelines and electronic media education. |
| Jahanzeb Malik et al. 2020  [19] | Rawalpindi | Benazir Bhutto Hospital/ Outpatient department | UTI | E. coli=330 (75%)  In females=223 (51%)  In males=107 (24%) | 8 months | 440 | Urine | Culture-positive samples:  In males=144  In females=296 | AMR is a global issue, especially in Pakistan, where mild symptoms often prompt an urgent response to over-prescription of antimicrobial agents. |
| Mohsin Khurshid et al. 2020  [73] | Lahore | Five TCHs/ ICU, medical, oncology, surgical, urology, pediatric and cardiology ward | Nosocomial infection | A. baumannii isolates=156 (100%)  Carbapenem-resistant isolates=139 (89.1%)  Acinetobacter baumannii  blaOXA-23-like genes=136 | June to November 2017 | 156 | Documented infections: Respiratory=68 (43.6%)  Bloodstream=30 (19.2%)  CNS=7 (4.5%)  UTI=22 (14.1%)  Other=29 (18.6%) | ICU=44 (28.2%) Medical=40 (25.6%)  Oncology=27 (17.3%) Surgical=24 (15.4%) Urology=11 (7.1%) Paediatric=9 (5.8%) Cardiology=1 (0.6%) | This study describes the molecular epidemiology of carbapenem-resistant A. baumannii isolates in Pakistan. Doxycycline showed the highest sensitivity. |
| Mohsin Khurshid et al. 2020  [39] | Lahore | TCH/ NM | Infected Wounds | Acinetobacter isolates=204 (100%) | During 2017 and 2018 | 204 | Wound | Out of 204 isolates,  MDR= 202 (99%)  XDR= 178 (87%) | The continuous surveillance of antimicrobial resistance, and strict adherence to infection control guidelines are crucial for reducing future major outbreaks. |
| Madiha Khan et al. 2020  [66] | Lahore | TCH/ NM | Liver and kidney patients | S. aureus=253 (12.75%)  MRSA=39 (15.41%) | April 2019 to April 2020 | 1984 | Urine, folly’s tip, pus, blood, sputum and wound swab | S. aureus=253/1984  MRSA=39/253 (15.41%) | Pakistan's limited data on MRSA colonization in patients with liver and kidney disease underscores the need for more research for effective control and monitoring. |
| Hizbullah Jan et al. 2020  [74] | Peshawar | Khyber Teaching Hospital/ Surgical B unit | Patients with a surgical site infection | Gram positive isolates=130 (48.1%)  Gram negative isolates=140 (51.9%) | March 2017 to March 2018 | 357 | Culture reports | Culture positive=270 (75.6%)  Staphylococcus aureus was isolated in 48.1% cases followed by:  E. coli=23.3%  Pseudomonas=10.7%  Enterobacter=10.4%  Citrobacter=4.4%  Proteus=1.9%  Klebsiella=1.1% | Antibiotic resistance is rapidly gaining traction, necessitating regular surveillance studies and susceptibility testing to limit MDR. |
| Tariq Mahmood et al. 2020  [32] | Multan | Nishtar medical university and hospital/ Orthopedic Surgery and emergency department | Patients with open fractures of the extremities | S. aureus=112 (57.4%)  Streptococcus=34 (17.4%)  E. coli=22 (11.3%)  MRSA=5 (2.5%)  Pseudomonas=14 (7.2%) | 29 June 2019 to 5 July 2020 | 195 | Wound swabs | Culture positive=187 (95.8%)  No growth=8 (4.1%) | Open extremity fractures often have positive bacteria cultures, most commonly gram-positive. Early debridement and skeletal stabilization are recommended. |
| Muhammad Imran Khan et al. 2020  [20] | Rawalpindi | Holy Family Hospital/ Pathology department | UTI | MDR bacterial isolates:  E. coli=23 (69.7%)  K. pneumoniae=7 (21.2%)  P. aeruginosa=3 (9.1%) | - | 100 | Urine | Culture positive=33 (33%)  No growth=67 (67%)  E. coli infection in females=82.17% | The rapid transmission of MDR isolates by E. Coli and K. pneumoniae is leading to a global MDR era, necessitating stricter preventive measures and precise data calculation. |
| Zuhair Ali Rizvi et al. 2020  [4] | Rawalpindi | Benazir Bhutto Hospital/ Urology OPD | UTI | E. coli=410 (77.4%)  Klebsiella=34 (6.4%)  Enterobacter=32 (6%)  Pseudomonas=20 (3.8%)  Staphylococcus=18 (3.4%)  Proteus=8 (1.5%)  Citrobacter=6 (1.1%)  Morganella=2 (0.4%) | January 2017 to June 2017 | 1000 | Urine | Culture positive=530 (53%)  Culture negative=470 (47%) | E. coli, a common UTI pathogen, is causing antimicrobial resistance against commonly used antibiotics, necessitating proper guidelines and research on appropriate antibiotic prescription and usage in UTI treatment. |
| Aftab Durrani et al. 2020  [21] | Hyderabad | Diagnostic and research laboratory/ Outpatient department of healthcare units | Typhoid fever | MDR Salmonella Typhi=92 (100%) | November 2016 to November 2017 | 92 | Blood | Patient samples=100% positive for polyvalent antisera for O antigen | In Sindh, MDR salmonella infection is common. To deal with this evident concern, practice guidelines should be built. |
| Mohsin Khurshid et al. 2020  [70] | Lahore | Five TCHs/ NM | Nosocomial infection | Non-duplicate Acinetobacter baumanni isolates=143 (100%) | January to July 2017 | 143 | Tracheal secretions=32,  Blood=26, Sputum =22, Urine=16, Pus=14, Wound swab=10,  CSF=7, Bronchial washings=6,  ETT=5,  Catheter tip=3,  Fluid=2 | Non-susceptible isolates to at least one of the tested aminoglycosides including amikacin and gentamicin= 133 (93%) | Because different antibiotics are used in different clinical situations, the study shows that aminoglycoside-resistant A. baumannii is heterogeneous in Pakistani hospitals. |
| Ghulam A. Maka et al. 2020  [40] | Hyderabad, Sindh | The diagnostic and research laboratory/ NM | Upper Respiratory tract infection (URTI) | P. aeruginosa=29 (48.33%)  K. pneumoniae=27 (45%)  E. coli=4 (6.67%) | August 2015 to March 2016 | 201 | Sputum and throat swab | Bacterial isolates belonging to Gram-negative group only=60 (29.85%) | Although antibiotic resistance is rising and amikacin is effective against Gram-negative bacteria that cause upper respiratory tract infections, empirical therapy should still utilize antibiotics sparingly. |
| Mehmood Hussain et al. 2021  [46] | Rawalpindi | Pak Emirates Military Hospital and Armed Forces/ Nephrology department | Catheter related Blood stream infection | CoNS=39  S. aureus=29  S. haemolyticus=6  E. faecium=2  K. pneumoniae=18  A. baumannii=17  P. aeruginosa=9  E. coli=6  Serratia Marcescens=4 | May 2019 to April 2020 | 753 | Blood and catheter tip | Culture positive=136 (18.06%)  Gram positive infection=76 (55.88%)  CoNS=28.68%  S. aureus=21.32%  Gram negative infection=60 (44.11%)  K. pneumoniae=13.24%  A. baumannii=12.50% | Over 80% of CKD patients require hemodialysis via non-tunneled catheters, but this increases risk of CRBSI, a 25% mortality rate. IJV catheters are preferred due to low infection rates. |
| Hasan Ejaz et al. 2021  [62] | Lahore | TCH/NM | Admitted patients with non-duplicate A. baumannii clinical isolates | CR-AB=113/174 (64.9%)  Harbored blaOXA-23 and blaNDM-1=49.5% and 24.7% respectively  A total of 11 (9.7%) isolates co-harbored blaOXA-51, blaNDM-1, and blaOXA-23 | September 2020 to December 2020 | 174 | Sputum=81 (46.5%)  Pus=42 (24%)  Blood=19 (10.9%)  Urine=18 (10.3%)  Throat swab=7 (4%)  CSF=3 (1.7%)  Nasal swab=2 (1.1%)  Seminal fluid=1 (0.6%)  HVS=1 (0.6%) | Male=64.9%  Female=35.1%  ICU=131 (75.2%)  Male surgical ward=13 (7.4%)  Female medical ward=11 (6.3%)  OT=8 (4.6%)  Male medical ward=6 (3.4%)  Female surgical ward=5 (2.8%) | According to the study, CR-AB ST2 clones carrying blaOXA-23 and blaNDM-1 are common in Pakistani clinical settings, which may lead to a higher death rate.  The National Action Plan on Antimicrobial Resistance must be strictly followed in order to lessen the effects of these strains. |
| IRFAN AHMAD et al. 2021  [5] | Rahim Yar Khan | Sheikh Zayed Medical College/Hospital/ Medical department | Cirrhotic patients with spontaneous bacterial peritonitis | E. coli=22 (62.9%)  Klebsiella=3 (8.6%)  Gram positive cocci=2 (5.7%)  Streptococcus=3 (8.6%)  S. aureus=4 (11.4%)  P. aeruginosa=1 (2.8%) | 20 March to 23 November 2019 | 35 | Ascitic fluid | Culture positive=35 (100%)  Culture positive neutrocytic ascites=24 (68.6%)  Culture positive non-neutrocytic (bacterascites) ascites=11 (31.4%) | E. coli is the primary cause of SBP, with Gram positive bacteria accounting for 25% of cases, indicating the need for individualized antibiotic treatment. |
| Shama Hashmani et al. 2021  [68] | Hyderabad | Diagnostic and Research laboratory of Liaquat University of Medical & Health Sciences/ NM | Bacterial Infection | CoPS=97 (62.1%)  CoNS=59 (37.9%) | October 2018 to September 2019 | 160 | Blood, pus and wound | Staphylococcal isolates=156 | Usually, Hyderabad showed higher levels of beta-lactam antibiotic resistance in Staphylococcal isolates than other regions of Pakistan where resistance has previously been reported. |
| Sundas Ishtiaq et al. 2021  [33] | Rawalpindi | Fauji Foundation Hospital/ Surgery department | Surgical site infection | S. aureus=27 (21.09%)  MRSA=26 (20.31%)  E. coli=24 (18.75%)  P. aeruginosa=18 (14.06%)  A. baumanii=11 (8.59%)  E. fecalis=7 (5.47%)  K. pneumonia=5 (3.91%)  M. morganii=1 (0.87%)  Bacillus subtilis=1 (0.87%)  P. agglomerans=1 (0.87%)  VRE=1 (0.87%) | 7 May 2018 to 7 November 2018 | 128 | Pus specimens | Culture positive=128 (100%) | Surgical site infections are most commonly caused by S. aureus, E. Coli, MRSA, and Pseudomonas, with Cloxacillin and Augmentin being the most resistant antibiotics. |
| Basit Zeshan et al. 2021  [47] | Lahore | TCH/ NM | COVID-19 Patients with Comorbidities | S. aureus=24 (7%)  S. agalactiae=6 (1.75%)  E. coli=98 (28.65%)  K. pneumonia=84 (24.55%)  P. aeruginosa=51 (14.91%)  A. baumannii=49 (14.32%)  S. maltophilia=5 (1.46%)  Citrobacter freundii=4 (1.16%)  Serratia=4 (1.16%)  Proteus=3 (0.87%) | 2 August 2021 to 31 October 2021 | 856 | Sputum=165  Tracheal aspirate=156  Bronchoalveolar lavage=117  Pleural fluid=3  Urine=238  Wound=102  Blood=60  FCT=6  Pus=6  Abscess=3 | Culture positive=342 (39.95%)  Gram-positive bacteria=39 (11.40%)  Gram-negative bacteria=303 (88.59%) | The study highlights high AMR rates in COVID-19 patients with specific comorbidities. This makes it difficult to prioritize treatment. Antibacterial usage policies, periodic audits, and systematic efforts are required. |
| Nureen Zahra et al. 2021  [57] | Lahore | Lahore General hospital (GLH), Mayo hospital (MH) and Jinnah hospital (JH)/ Surgical ICU | Surgical Intensive Care Unit Patients | A. baumannii=90 (15.177%) | June 2017 to March 2019 | 593  (GLH=396  MH=103  JH=94) | Wound, blood, burn and pus | Culture positive=90 (15.177%)  Wound=16 (17.7%)  Blood=19 (21.1%)  Pus=27 (30%)  Burn=28 (31.1%) | This study investigates the treatment of drug-resistant A. baumannii and the connection between β-lactamases and phenotypic resistance patterns. |
| Hamza Ali Khan et al. 2021  [24] | Peshawar | Kyber teaching hospital/ NM | UTI | E. coli=91 (61.5%) | 20th February to 19th August 2018 | 148 | Urine | Females=81 (54.73%)  Males=67 (45.27%) | A reassessment of UTI treatment and routine monitoring of antimicrobial susceptibility in community and hospital settings are required due to nitrofurantoin resistance in E. coli isolates. |
| KIRAN FATIMA et al. 2022  [48] | Rawalpindi | Holy Family Hospital/ ICU and general departments such as surgical, medical allied ward | ICU patients | Acinetobacter=141 (69.1%)  Klebsiella=32 (15.7%)  E. coli=15 (7.4%)  S. aureus=8 (3.9%)  Pseudomonas=6 (2.9%)  Proteus=1 (0.5%)  Coliform=1 (0.5%) | Between July 2019 and January 2020 | 204 | ETT, wound, pus, urine, CVP, sputum, pleural fluid and foleys catheter | Frequency of Acinetobacter in:  Medical ICU=56 (27.4%)  Surgical ICU=33 (16.1%)  Burn ICU=10 (4.9%)  Pediatric ICU=3 (1.4%) | Hospitals must implement strict infection control measures, maintain antibiotic use, implement protocols, and enhance hand hygiene to prevent A. baumannii transmission. |
| Hasan Ejaz et al. 2022  [63] | Lahore and Faisalabad | Laboratories of the tertiary care hospitals | Patients in ICU and medical wards | Acinetobacter calcoacetius-baumannii complex=455  CRACB cases=200  CSACB cases=255 | November 2020 to May 2021 | 1985 | Clinical samples (pus, tracheal swabs, blood, CSF and urine)  Pus=35.4%  Tracheal secretions=23.5% | Culture positive=1250 (62.9%)  CRACB cases=200 (43.9%)  All isolates exhibited the blaOXA-51 gene | The study finds co-occurring genes and CRACB, a bacterium resistant to the majority of antibiotics, which raises public health concerns and necessitates national AMR surveillance and collaborative initiatives. |
| Ansar Abbas et al. 2022  [67] | Lahore | Fatima Memorial Hospital/ Pathology department | Infected patients | S. aureus=400 (40%)  S. pyogenes=50 (5%) | - | 1000 | Blood, pus, swabs, sputum, urine, fluid and semen | Gram positive isolates= S. aureus (40.0%) and S. pyogenes (5.0%) | There is a concerning increase in resistance to nearly all antibiotics, with few exceptions, indicating a concerning trend. |
| Muhammad Kamran et al. 2022  [59] | Punjab region | Two government-owned and two privately-owned hospitals/ NM | HAI | P. aeruginosa=39 (45.8%) | **-** | 170  (85 from clinical site) | Skin surfaces=17  Sputum=17  Urine=17  Blood=17  Wound=17 | Wound samples =28.2 %  Urine samples=12.8 % | P. aeruginosa is resistant to anti-pseudomonal drugs, and contamination from high-risk sources asks about inherent gene transfer in natural microbial ecosystems. |
| RASHEED AHMED BHATTI et al. 2022  [34] | Jamshoro and Larkana | Liaquat University of Medical and Health Sciences and SMBBMU/ Department of Orthopedic surgery | Patients with Open Tibial Fracture | S. Aureus=60 (40%)  MRSA=21 (14%)  Enterococcus=6 (4%)  B-Streptococcus=6 (4%)  P. aeruginosa=12 (8%)  Acinetobacter=6 (4%)  E. coli=24 (16%)  Klebsiella=15 (10%) | 14th October 2015 to 13th April 2016 | 150 | Wound swab | Culture positive=150 (100%) | There are higher infection rates in open fractures, primarily at bone extremities, most commonly with S. aureus. Antibiotic resistance and sensitivity are assessed. |
| ALMAS RAZA et al. 2022  [25] | Lahore | Pak Red Crescent Teaching hospital/ Pathology department | Bacteremia | E. coli=8 (12%)  Klebsiella=6 (9%)  Enterobacter cloacae=2 (3%)  Salmonella=29 (45%)  Proteus=1 (2%)  Pseudomonas=11 (17%)  Acinetobacter=7 (10%)  Serretia=1 (2%) | November 2019 to October 2020 | 1100 | Blood | Gram negative rods=65 (6%)  Culture positive=65 | The percentage of Gram-negative rods in blood specimens was only 6%. Among the E. coli positive cultures, Amikacin was the most sensitive antibiotic. |
| Uzma Noor et al. 2022  [26] | District Swat | Anwar clinical laboratory Saidu Sharif/ NM | UTI | E. coli=115 (69.6%)  K. pneumoniae=31 (18.7%)  Citrobacter=19 (11.5%) | - | 200 | Urine | Culture positive= 165 (82%)  E. coli=115  MDR positive=36 (31.3%)  MDR negative=79 (68.6) | E. coli was the most prominent bacterial isolate in urine samples, with ampicillin resistance being the most observed. MHT was detected in any isolate. |
| MUHAMMAD ASIM RANA et al. 2022  [6] | Lahore | Bahria International Hospital and Farooq Hospital/ Outpatient department | UTI | E. coli=133 (70%)  Klebsiella=34 (17.9%)  Enterococcus=17 (8.9%)  Acinetobacter=6 (3.2%) | 1st January 2019 to 31st December 2020 | 190 | Urine | Complicated UTI=66  Uncomplicated UTI=124  E. coli positive in Complicated UTI=47 (71.2%)  E. coli positive in Uncomplicated UTI=86 (69.3) | UTIs significantly burden the economy and public health, affecting individual quality of life. E. coli and Klebsiella cause most cases, with antibiotics becoming ineffective due to resistance. |
| ZAHRA IMDAD et al. 2022  [7] | Lahore | The Children’s Hospital & Institute of Child Health/ Emergency of Department of Paediatric Medicine | Chronic Lung Disease | H. parainfluenza=59 (36.9%)  H. influenza=69 (43.1%)  S. pneumoniae=32 (20%) | 2020-2021 | 160 | Sputum | Culture positive=160 (100%) | Children with chronic lung disease are most frequently isolated by H-influenza with H-parainfluenza showing higher antibiotic sensitivity. |
| Irum Mahmood et al. 2022  [43] | Rawalpindi | Armed forces Institute of Ophthalmology/ NM | Patients with Blepharitis | CoNS=77 (48.1%)  S. aureus=71 (45%)  S. pneumoniae=11 (6.9%) | October 2019 to October 2021 | 160 | Soaked swabs were rubbed against the base of eyelashes | Anterior blepharitis=62 (38.7%)  Posterior blepharitis=98 (61.3%) | Blepharitis is primarily caused by CoNS and S. aureus, which exhibit significant sensitivity to chloramphenicol, Gentamycin, Amoxicillin-Clavulanate, and Cotrimoxazole. |
| Syed Bilal Hafeez et al. 2022  [58] | Islamabad | Shifa International Hospital/ Emergency Medicine | Catheter-Related Bloodstream Infection | E. coli=41 (25%)  P. aeruginosa=123 (75%) | 4 March 2022 and 4 August 2022 | 164 | Blood, sample taken from hub of catheter and peripheral vein | Patients with positive CRSBI=20 (12.12%) + 68 (41.5%)  Patients with catheter colonization=68 (41.5%)  Culture negative=46.3% | The study emphasizes the importance of regular monitoring of the microbiological spectrum in ICUs, particularly for E. coli and P. aeruginosa, which are resistant to modern antibiotics. |
| Muhammad Mubashar Idrees et al. 2022  [8] | Multan | MIKD hospital and IMBB, BZU/ NM | UTI | E. coli=293 (51.2%)  Klebsiella=88 (15.4%)  Enterococcus=88 (15.4%)  Pseudomonas=54 (9.4%)  S. aureus=18 (3.2%)  CoNS=17 (3.0%)  Proteus=14 (2.5%) | September 2020 to December 2020 | 1000 | Urine | Culture positive=600 (60%)  Uropathogens detected in=572 (57%)  Rejected samples (contaminated of S. epidermidis) =28 | Drug resistance in developing countries like Pakistan is escalating due to overuse and misuse of antibiotics like Ampicillin, Augmentin, and Co-trimoxazole for treating UTIs. |
| Bashir Aqib and Rehman Bilal et al. 2022  [27] | Peshawar | Rehman Medical Institute/ Pathology department | UTI | MDR E. coli positive=48/150 (32%)  MRD E. coli negative=101/150 (67.3%) | December to April 2021 | 800 | Urine | UTI positive samples=150 (18.75%) | Among other reasons, self-medication, incomplete medication, and excessive use are contributing to the rise in MDR antibiotic-resistant E. Coli. |
| IRUM ANWAR et al. 2022  [9] | Rawalpindi | Holy Family Hospital/ Microbiology department | UTI | E. coli=110 (40.7%)  Candida=76 (28%)  Enterococcus=29 (10.6%)  Klebsiella=26 (9.8%)  Coliform=12 (4.3%)  Acinetobacter=5 (1.97%)  S. aureus=4 (1.58%)  P. aeruginosa=8 (2.76%) | 6 months (January to May) | 1000 | Urine | Significant bacteriuria=270 (27%)  No growth=730 (73%) | Antibiotic sensitivity changes rapidly, especially in developing nations, due to inappropriate prescribing, over-the-counter purchases, and short treatment durations, leading to resistance. |
| Yaseen Anwar et al. 2022  [49] | Peshawar | Hayatabad Medical Complex/ Outpatient department, ENT, emergency, paeds, gynae and endocrinology department | Bacterial infections | Gram-positive isolates=40 (38.1%)  Gram-negative=65 (61.90%) | May 2017 to October 2017 | 753 | Pus=31 (29.5%)  Urine=40 (38%)  Swab=34 (32.3%) | Culture positive=105 (13.94%)  Most prevalent gram-positive=E. coli=30 (29%)  Most prevalent gram-negative=S. epidermidis=10 (9.52%) | The study found E. coli (29%), S. aureus (19.4%), and P. aeruginosa (13.33%), with higher incidence in urine samples. Resistance to antibiotics was highest in gram-negative isolates. |
| Erum Afzal et al. 2022  [28] | Multan | CHICH/ Department of developmental and behavior pediatrics | UTI in children with cerebral palsy | E. coli=10 (26.3%)  Klebsiella=4 (10)  Enterobacter=2 (5.3%)  Pseudomonas=2 (5.3%) | September 2020 to September 2021 | 52 | Urine | No growth=20 (52.7%)  UTI=38 (73.1%)  USG findings:  Positive=24 (63.1%)  Negative=14 (23.8%) | UTI is a frequent cause of renal compromise in children with cerebral palsy. Early detection and care are therefore necessary to avoid complications and antibiotic resistance. |
| Muhammad Kamran et al. 2022  [64] | Islamabad | Department of Biosciences, Comsats University | Patients with suspected E. coli infection in hospitals of Rawalpindi & Islamabad | Urine=the most prominent source of E. coli (56.2%) followed by:  Blood=19%  Pus=8.3%  CSF=5.8% | - | 121 | Urine=68 (56.2%)  Blood=23 (19%)  Pus=10 (8.3%)  CSF=7 (5.8%)  Tracheal secretion=5 (4.1%)  Sputum=4 (3.3%)  Fluid=2 (1.7%)  Bronchial washings=1 (0.8%)  ETT=1 (0.8%) | ESBL negative=74 (61.2%)  ESBL positive=47 (38.8%)  ESBL genes:  blaCTX-M  blaTEM  blaSHV | The current study shows the extent of antibiotic resistance and the underlying genes, particularly the blaCTX-M gene, in the isolates of E. coli. |
| Aftab Ali et al. 2022  [10] | KPK | District hospital and some private laboratories of district Buner/ Outpatient department | Patients visiting hospital for fever | Salmonella typhi=98 (40.8%)  S. aureus=6 (0.025%)  Acinetobacter=3 (0.012%)  E. coli=2 (0.008%) | Jan 2020 to May 2021 | 240 | Blood | S. typhi positive=98 (40.8%)  MDR=28 (28.6%)  XDR=17 (17.4%)  Non-MDR/XDR=53 (54.0%) | The study emphasizes the necessity for cautious medicine prescription, surveillance investigations, and the availability of typhoid vaccination due to the increasing resistance of S. typhi to antimicrobial drugs. |
| Muhammad Ashraf Hussain et al. 2023  [11] | Kharian | CMH/ Neonatal intensive care unit | NICU patients with Neonatal sepsis | S. aureus=77 (33.9%)  MRSA=48  MSSA=29  K. pneumoniae=33 (14.5%)  CoNS=28 (12.3%)  A. baumannii=19 (8.4%)  Stenotrophomonas maltophilia=7 (3.1%)  P. aeruginosa=6 (2.6%)  E. faecalis=5 (2.2%)  Enterobacter cloacae=4 (1.8%)  K. oxytoca=3 (1.3%) | January 2019 to December 2020 | 4892 | Venous blood | Clinically diagnosed neonatal sepsis cases=342  Culture positive=227 (66.4%)  Gram-positive=59.5%  Gram-negative=40.5% | S. aureus is a common cause of sepsis in newborns, and because of its high methicillin resistance, antibiotic resistance must be strictly controlled. |
| JAIS KUMAR KARMANI et al. 2023  [12] | Islamabad | Dr. Akbar Niazi Teaching Hospital/ Department of general medicine and nephrology | Lower UTI | E. coli=77 (84.6%)  Klebsiella=7 (7.7%)  Enterococcus=6 (6.6%)  P. aeruginosa=1 (1.1%) | January 2019 to January 2020 | 206 | Urine | Positive urine culture=91 (44.2%) | Antibiotic resistance, primarily by E. coli, is rapidly advancing. Continuous surveillance, deeper drug resistance research, and health education are necessary. |
| Muhammad Musarrat Jamal et al. 2023  [13] | Sialkot | Islam Teaching Hospital, Children Complex Hospital, and Islam Central Hospital/ Department of pediatric | UTI | E. coli=159 (64.1%)  Klebsiella=24 (9.6%)  Proteus=23 (9.2%)  Pseudomonas=12 (4.8%)  Enterobacter=9 (3.6%)  Acinetobacter=7 (2.8%)  Citrobacter=2 (0.8%)  Enterococcus=7 (2.8%)  CoNS=5 (2.01%) | January to December 2020 | 248 | Urine | Gram negative Bacilli=236 (95.2%)  Gram positive Bacilli=12 (4.8%) | High resistance to pathogens is due to inappropriate infection control and antibiotic misuse, necessitating regular reevaluation of guidelines and surveillance for emerging resistance. |
| FATIMA KHALID et al. 2023  [69] | Lahore | Shalamar Teaching Hospital/ Pathology Department | MRSA infection | S. aureus=149 (100%) | December 2020 to May 2021 | 149 | Nasal swab, blood, urine, pus, sputum | Out of 149 clinical isolates:  Pus=52 (34.9%)  Wound=49 (32.9%)  Blood=10 (6.7%)  Others=38 | There is high prevalence of MDR in MRSA, particularly in Shalamar region. Further research is needed |
| Farida Sherazi et al. 2023  [14] | Peshawar | Naseer Teaching Hospital/ Pediatric department | Fever without Localizing Signs (FWLS) | Salmonella typhi, Paratyphi and Gallinarum=71 (42.77%) | October 2019 to October 2021 | 166 | Blood | Salmonella isolated patients=71 (42.77%)  Not detected=95 (57.22%) | MDR and XDR are major threats in treating typhoid, with trimethoprim being the most resistant antibiotic. |
| Ali Akhtar et al. 2023  [50] | Lahore | The Children’s hospital and The Institute of Child Health/ Cancer ward | Pediatric cancer patients | S. aureus=177 (59%)  S. pyogenes=69 (23%)  Streptococcus=3 (1%)  S. pneumoniae=48 (16%)  S. viridens=3 (1%)  Pseudomonas=102 (34%)  E. coli=108 (36%)  Klebsiella=90 (30%) | 6 months | 300 | Blood=29%  Urine and mucosal swabs | Gram positive isolates=300  Gram-negative isolates=300 | Antibiotic resistance poses a significant threat to chemotherapy, necessitating a government-level policy involving experts to control AMR and improve survival rates. |
| Altaf Hussain et al. 2023  [35] | Karachi | Jinnah Postgraduate Medical Center/ Emergency department | Patients with open fractures of the extremities | S. aureus=102 (59.302%)  Streptococcus=31 (18.02%)  MRSA=6 (3.48%)  E. coli=19 (11.04%)  Pseudomonas=14 (8.13%) | November 2021 to November 2022 | 180 | Wound swabs | Culture positive=172 (95.55%)  No growth=8 (4.44%) | Most open extremity fractures have positive bacterial cultures, most commonly gram-positive. Patients should undergo early debridement and skeletal stabilization. |
| FATIMA IQBAL et al. 2023  [15] | Rawalpindi | Wapda Hospital/ Outpatient department | UTI among children | E. coli=43 (60.6%)  Klebsiella=11 (15.5%)  Enterococcus=5 (7%)  E. cloacae=4 (5.6%)  P. aeruginosa=4 (5.6%)  Proteus=2 (2.8%)  S. saprophyticus= 2 (2.8%) | June 2022 to November 2022 | 176 | Urine | Culture positive=71 (40.34%)  Gram-negative=62 (87.3%)  Gram-positive=7(9.8%) | Amikacin is a sensitive, cost-effective parental drug, necessitating regular regional studies for updated treatment protocols. |
| Salma Zeb et al. 2023  [16] | Peshawar | Lady Reading Hospital/ Medicine department | UTI-symptomatic stroke patients | E. coli=45 (56.25%)  S. saprophyticus=15 (18.75%)  Klebsiella=10 (12.5%)  Enterobacter=5 (6.25%)  Proteus=5 (6.25%) | September 2022 to February 2023 | 80 | Urine | Culture positive=80 (100%) | Stroke prevalence underscores awareness. Antibiotic resistance patterns indicate varied drug use, emphasizing the need for targeted therapy. |
| Mohammad Riaz Khan et al. 2023  [29] | Peshawar | Khyber Teaching Hospital and Hayatabad Medical Complex/ NM | Septicemia | E. coli=184 (41.5%)  P. aeruginosa=78 (17.6%) | - | 3389 | Blood | Culture positive=443 (13.07%)  Gram-negative=262/443 (59.1%)  Gram-positive=181/443 (40.8%) | Antibiotic resistance is escalating, necessitating a rationalization of treatment strategies, leading to pan-drug resistance and the need for new antimicrobial drugs. |
| Ummara Altaf et al. 2023  [51] | Lahore | Ghurki Trust Teaching Hospital/ Orthopedic, ICU, medical, surgical, paeds and gynecology | Infected patients | E. coli=140 (28.1%)  P. aeruginosa=53 (10.6%)  K. pneumoniae=50 (10%)  A. baumannii=35 (7%)  Proteus=31 (6.2%)  MSSA=125 (25.1%) | May 2018 and December 2018 | 465 | Pus=272 (58.5%)  Urine=86 (18.5%)  Tissue=50 (10.8%)  Catheterization=21 (4.5%)  Bone=18 (4%)  Blood=10 (2.1%) | Detected isolates from 465 patients=497 (as 32 patients had polymicrobes)  Culture positive=465 | Culture sensitivity reports reduced antibiotic utilization. However, concerns about AMR and IV administration led to the need for ASP implementation and antibiotic guidelines. |
| Zeeshan Taj et al. 2023  [60] | Lahore, Multan, Peshawar and Islamabad | 5 TCHs:  A, B (Lahore)  C (Peshawar)  D (Islamabad)  E (Multan)/ ICU, medicine, surgery and other departments | HAI | A. baumannii=130 (100%) | January 2020 to July 2021 | 130 | Tracheal tube= 30  Blood= 25  Sputum= 21  Urine= 12  Pus= 15  Wound swab= 8  Bronchi=6  CSF=4  ETT=4  Catheter tip=4  Fluid=1 | Patient’s frequency in:  ICU=43 (33.08%)  Medicine=29 (22.31%)  Surgery=20 (15.38%)  Frequency in 5 TCHs:  A=36 (27.69%)  B=32 (24.62%)  C=12 (9.23%)  D=36 (27.69%)  E= 14 (10.77%) | The study reveals a sequence-specific distribution of biocide and antibiotic resistance genes in A. baumannii isolates in Pakistan. High resistance rates were observed. |
| Nasreena Bashir et al. 2023  [52] | Peshawar | The Khyber teaching hospital, The Hayat Abad medical complex and The North West general hospital/ Nephrology, endocrinology and pulmonology unit | Infected patients mainly UTI | E. coli=32 (29.35%)  S. aureus=17 (15.59%)  Klebsiella=14 (12.84%)  Enterobacter=10 (9.17%)  Citrobacter=9 (8.25%)  Acinetobacter=6 (5.50%)  Pseudomonas=5 (4.58%)  Proteus=5 (4.58%)  Others=11 (10.09%) | August 2021 to February 2022 | 130 | Urine, blood, CSF, wound swabs, pus and sputum | Culture positive=109 (83.84%):  Gram negative bacteria=92/109  Gram-positive bacteria=17/109 | MDR pathogens pose a significant health issue. To save antibiotic efficacy, hospitals should implement effective stewardship programs and acquire clinical pharmacist services to guide rational antibiotic use practices. |
| NIDA BASHARAT KHAN et al. 2023  [23] | Faisalabad | Private and Government hospitals/ NM | Urogenital Tract Infections | E. coli=132 (73.3%) | - | 180 | Midstream urine | Culture positive=132 (73.3%)  Culture negative=48 (26.6%) | E. coli's resistance to antimicrobial drugs necessitates antimicrobial susceptibility testing for UTI treatment to prevent increasing resistance. |
| Ambreen Ahmad et al. 2023  [72] | Peshawar | Hayatabad Medical Complex/ Pediatrics department | Pediatric patients with clinical features of enteric fever | Salmonella typhi=104 (100%) | 1st April 2021 to 30th June 2022 | 104 | Blood culture reports | Female children=32  Male children=72 | Salmonella typhi's sensitivity to antibiotics is decreasing, necessitating strict antimicrobial use surveillance to avoid further worsening of antibiotic efficacy in Salmonella infection treatment. |
| Rabia Ali et al. 2023  [17] | Faisalabad | Independent University Hospital/ Outpatient department | Community acquired UTI | E. coli=21 (66%)  Klebsiella=5 (16%)  Pseudomonas=3 (9%)  Staphylococcus saprophyticus=3 (9%) | July 2021 to July 2022 | 64 | Urine | Positive bacterial culture=32 (50%)  Negative bacterial culture=32 (50%) | Over-prescription of antibiotics in clinics rises MDR bacteria prevalence, causing community-acquired UTI. Good antibiotic stewardship practices can prevent drug-resistant bacteria emergence. |
| Shabir Ahmad et al. 2023  [42] | Peshawar | Hayatabad Medical Complex/ NM | Wound and surgical infection | P. aeruginosa=62 (62%) | - | 100 | Wound pus | Of 100 samples,  P. aeruginosa=62 | All combinations, except selected antibiotics, showed synergism against MDR P. aeruginosa. Further studies are needed. |
| Abdul Haseeb et al. 2023  [56] | Lahore | Ghurki Trust Teaching Hospital/ Orthopedic ward | Patients with positive culture reports of E. coli and MSSA microbes | E. coli=118 (46.1%)  MSSA=138 (53.9%) | September 2020 to December 2020 | 256 | Pus, urine, tissue, dead bone, blood, tracheal secretion and CSF | Majority of patients= surgical site infection (59.4%) | Sensitivity reports help limit antibiotic therapy, necessitating de-escalation practice for medical and surgical patients, and guidelines and limiting healthcare professionals' prescribing authority are also needed. |
| Aiman Waheed et al. 2023  [37] | Peshawar | Khyber Teaching Hospital and Hayatabad Medical Complex/ Dermatology department | Community acquired superficial skin infections | S. aureus= 50 (31.7%)  P. aeruginosa=32 (20.25%)  E. coli=31 (19.62%)  Streptococcus=24 (15.18%)  Enterobacter=21 (13.3%) | July 2019 to October 2019 | 205 | Swab samples from the infected skin area | Total culture positive=158 (77.07%)  No growth=54 (26.34%)  Single isolate growth=142 (94%)  Multiple growth=9 (6%) | Doxycycline is recommended as the antibiotic for S. aureus, as Gentamicin and Amikacin have shown good susceptibility to both Gram positive and Gram-negative isolates. |
| Ghulam Abbas et al. 2023  [36] | Peshawar | Khyber Teaching Hospital/ Outpatient department and Medical and surgical unit | Diabetic foot ulcers | E. coli=99 (45.8%)  P. aeruginosa=49 (22.7%)  Proteus mirabilis=18 (8.3%)  MRSA=21 (9.7%)  MSSA=15 (6.9%)  Acinetobacter=4 (1.9%)  K. pneumoniae=1 (0.5%)  Proteus vulgaris=4 (1.9%)  Strep faecalis=5 (2.3%) | January 2019 to September 2020 | 238 | Deep wound swabs | Positive culture=216 (90.75%)  Gram-positive isolates=175 (81%)  Gram-negative isolates=41 (19%)  Pus culture positive=215 (91%)  Pus culture negative=23 (9%) | Amikacin is effective well against S. aureus and gram-negative rods, but improper use might lead to an increase in antibiotic-resistant bacteria. It is advised to combine the right antibiotics for diabetic foot ulcers. |
| Husna Shams et al. 2023  [22] | Peshawar | Khyber Teaching Hospital/ Outpatient department | UTI | E. coli=52 (41.6%)  Enterobacter=45 (36%)  M. morganii=28 (22.4%)  ESBL positive=76 (60.8%)  MBL positive=77 (61.6%) | 13 August 2019 to 17 July 2019 | 200 | Urine | Culture positive=125 (62.5%)  No growth=75  ESBL negative=49 (39.2%)  MBL negative=48 (38.4%) | The study finds that urine samples contain E. Coli, Enterobacter, and M. morganii. Amikacin susceptibility rate is 62.4%. This suggests that policymakers and physicians should address antibiotic resistance in UTI patients. |
| GUL S et al. 2023  [44] | Peshawar | Lady Reading Hospital/ NM | Catheter-related Infection in Hemodialysis patients | S. aureus=13 (46.4%)  CoNS=11 (39.3%)  E. coli=2 (7.1%)  K. pneumoniae=1 (3.6%)  P. aeruginosa=1 (3.6%) | January 2021 and December 2021 | 200 | Catheters | Patients undergoing hemodialysis=152  S. aureus= 52 (34.2%)  CoNS= 47 (30.9%)  E. coli= 18 (11.8%)  K. pneumoniae= 11 (7.2%)  P. aeruginosa= 9 (5.9%)  Other gram-negative bacilli=15 (9.9%) | In hemodialysis patients, gentamicin may be an effective antibiotic for catheter-related infections; nevertheless, more research is required to improve treatment and prevention. |
| Muhammad Atif et al. 2023  [45] | Rawalpindi | Alsyed hospital/ NM | Indoor patients | E. coli=31 (16.14%)  K. pneumoniae=20 (10.41%)  K. oxytoca=4 (2.08%)  Serratia marcescens=5 (2.60%)  Proteus mirabilis=2 (1.04%)  Enterobacter cloacae=7 (3.64%)  Stenotrophomonas maltophilia=3 (1.56%)  Burkholderia cepacian=2 (1.04%)  A. baumannii=3 (1.56%)  P. aeruginosa=26 (13.54%) | - | 820 | ETT=321  Urinary catheter (Foley’s tip) =296 CVP tip=59  DJ catheters=123  [NG tube, suction tube, Parma catheter tube and drain tube] =21 | Microbial growth=192 (23.4%)  Urinary catheter=81 (27.4%)  ETT=64 (19.9%)  CVP line=19 (32.2%)  DJ tip=123 (13.8%)  Others=11 (52.4%) | The predominant cause of Pseudomonas infections associated with catheter use and other HAIs is Gram negative bacteria. Strategies for prevention and management need the use of potent antimicrobials, such as amikacin. |
| Rooh Ullah et al. 2023  [41] | Peshawar | MTI-LRH and MTI-KTH/ Burn Unit and Microbiology Department | Burn patients | P. aeruginosa=26 (16.25%) | - | 160 | Non-duplicate clinical Pus samples | P. aeruginosa positive:  Males=16 (61.53%)  Females=10 (38.46%) | Hospital antibiotic policies and antibiotic discipline are necessary due to the escalation of the problem caused by the lack of novel anti-pseudomonal medicines against MDR P. aeruginosa. |
| Zikria Saleem et al. 2023  [53] | Punjab region | Public and private sector microbiological laboratories | Patient culture reports with one particular resistant isolate | S. aureus=866 (34.3%)  E. coli=814 (32.2%)  P. aeruginosa=454 (18.0%)  K. pneumonia=269 (10.7%)  MDR isolates:  E. coli=606  P. aeruginosa=310  K. pneumonia=192  Proteus=56  A. baumannii=42  S. aureus=445  XDR isolates:  E. coli=2  P. aeruginosa=5  K. pneumonia=1 | January 2018- April 2019 | 2523 | Pus=1464 (58%)  Urine=718 (28.5%)  Blood=164 (6.5%)  Sputum=81 (3.2%)  Tissue=53 (2.1%)  Body fluids=43 (1.7%) | Gram-negative bacteria=65.4%  Gram-positive bacteria=34.3%  E. coli=497 (61.10%) was isolated from urine  S. aureus=704 (81.30%) and P. aeruginosa=307 (67.60%) were isolated from pus | The report recommends hospitals to adopt efficient antibiotic prescribing practices in order to lower AMR and pays attention to Pakistan's lack of antimicrobial therapies because of MDR microbes. |
| Nauman Khalid et al. 2023  [55] | Lahore | Ghurki Trust Teaching Hospital laboratory | Hospitalized patients | Acinetobacter=36 (8.5%)  Citrobacter=20 (4.7%)  E. coli=68 (16.1%)  Klebsiella=20 (4.7%)  Pseudomonas=41 (9.7%)  Proteus=30 (7.1%)  S. aureus=177 (41.9%)  S. epidermidis=11 (2.6%)  Strep=19 (4.5%) | January 2021 to June 2021 | 422 | Blood=8 (2%)  Wound=414 (98%) | Gram-positive isolates=207 (49.05%)  Gram-negative isolates=215 (50.94%) | Clinicians should employ updated antibiograms to assess bacterial susceptibility patterns, and culture sensitivity data help to rationalize the use of antibiotics in clinical practice while combating AMR. |
| Tassmia Afzal et al. 2024  [54] | Faisalabad | Department of Dermatology in Madinah Teaching Hospital | Patients with deep skin and soft tissue infection (SSTI) | Gram negative and positive microorganisms=1 (1.7%)  Gram negative microorganisms=30 (50%)  Gram positive microorganisms=29 (48.3%) | June 2023 to December 2023 | 60 | Pus/wound swab samples | Male=37 (61.7%)  Female=23 (38.3%) | Significant antibiotic resistance was observed in a trial of 60 individuals with deep skin and soft tissue infections; amikacin showed significant sensitivity and doxycycline high resistance, highlighting the necessity for focused treatment approaches. |
| Bibi Sazain Aman et al. 2024  [38] | Quetta | Bolan Medical Complex Hospital and the Sandeman Provincial Hospital | Patients with bacteria-infected skin | S. aureus=316 (39.5%)  Clostridium perfringens=18.96 (2.37%)  E. coli=120 (15.12%)  P. aeruginosa=98 (12.25%)  K. pneumonia=44 (5.5%) | June 2021 to May 2022 | 800 | Swab samples | Positive pathogenic bacteria=598 (74.7%)  Negative pathogenic bacteria=202 (25.25%)  Male-positive cases=380 (47.5%)  Female-positive cases=218 (27.25%) | Many bacterial infections can cause SSTIs, but S. aureus is the most prevalent. The necessity for cautious antibiotic use is highlighted by antibiotic resistance, particularly in S. aureus and P. aeruginosa. |
| Muhammad Saeed et al. 2024  [65] | Lahore | Jinnah Hospital | Admitted patients of all ages who were placed on a ventilator for at least 48hr before developing symptoms | B. cepacian=92 (68.7%)  B. multivorans=25 (18.7%)  B. cenocepacia=17 (12.7%)  B. cepacian=20 isolates from the ventilator tubing | June 2022 to May 2023 | 134 | Bronchoalveolar lavage=13 (9.7%)  Blood=72 (53.7%)  Bronchial washings=17 (12.7%)  ETT tip=9 (6.7%)  Endotracheal wash=6 (4.5%)  Tracheal secretion=17 (12.7%) | Male=85 (63.4%)  Female=49 (36.6%)  BCC (Burkholderia cepacia Complex) isolates=134 | The study calls for appropriate disinfection practices and new tubing in underdeveloped nations like Pakistan, highlighting the possible effects of BCC bacterial contamination in ventilators on critically ill patients. |

**AMR=**antimicrobial resistance**, CRBSI=** Catheter-related Blood stream infection, **CoNS=**Coagulase negative staphylococci**, CSF**=cerebrospinal fluid, **CVP** =central venous pressure, **ETT**=endotracheal tube, **FCT**= Foley’s catheter tip, **HAI**= hospital acquired infection, **HVS**=high vaginal swab, **ICU**=intensive care unit, **MBL**=Metallo beta-Lactamase **MRSA**=methicillin resistant staphylococcus aureus, **MSSA**=methicillin sensitive staphylococcus aureus, **NG**=nasogastric tube, **NM**=not mentioned, **NICU**=neonatal intensive care unit, **SBP**=spontaneous bacterial peritonitis, **TCHs**=tertiary care hospitals, **TS**=transferrin saturation, **UTI**=urinary tract infection.

**Supplementary Table S3:** Common microorganisms and Access group antibiotics in included studies

|  | **Access group antibiotics and their Susceptibility N (%)** | | | | | | | | | | | | | | |
| --- | --- | --- | --- | --- | --- | --- | --- | --- | --- | --- | --- | --- | --- | --- | --- |
| **Ref No.** | **Micro- organisms** | **AMC** | **AMK** | **AMP** | **AMX** | **CEX** | **CFZ** | **CHL** | **CLI** | **CLOX** | **CN** | **DO** | **NFT** | **SXT** | **T** |
| **[30]** | Salmonella typhi and Salmonella Paratyphi | **-** | **-** | 7 (13.5%) | - | - | - | 4 (7.7%) | - | - | - | - | - | 4 (7.7%) | **-** |
| **[61]** | Acinetobacter baumannii (n=59) | - | - | - | - | - | - | - | - | - | 6 (10.1%) | - | - | 19 (32.2%) | - |
|  | P. aeruginosa (n=47) | - | - | - | - | - | - | - | - | - | 15 (31.9%) | - | - | 35 (74.4%) | - |
|  | K. pneumonia (n=44) | - | - | - | - | - | - | - | - | - | 4 (9.1%) | - | - | 10 (25%) | - |
| **[1]** | E. coli | 20 (76.9%) | 19 (73.1%) | 10 (38.5%) | - | - | - | - | - | - | - | - | 22 (84.6%) | - | - |
|  | K. pneumoniae | 4  (50%) | 5 (62.5%) | 0  (0.0%) | - | - | - | - | - | - | - | - | 8  (100%) | - | - |
|  | Pseudomonas | 3  (50%) | 4 (66.7%) | 0  (0.0%) | - | - | - | - | - | - | - | - | 4 (66.7%) | - | - |
|  | Enterococcus | 0  (0.0%) | 1  (25%) | 4  (100%) | - | - | - | - | - | - | - | - | 4  (100%) | - | - |
|  | S. aureus | 1  (25%) | 4  (100%) | 1  (25%) | - | - | - | - | - | - | - | - | 4  (100%) | - | - |
| **[2]** | S. aureus | 47.1% | 85% | - | - | - | - | 68% | 66% | 28.3% | 5.6% | - | - | - | - |
|  | Enterococci | 0% | 100% | - | - | - | - | 0% | 100% | 100% | 0% | - | - | - | - |
|  | Pseudomonas | 7.4% | 85% | - | - | - | - | 14.8% | 7.4% | - | 32% | - | - | - | - |
|  | Klebsiella | 36.36% | 82% | - | - | - | - | 18.1% | 9% | - | 63% | - | - | - | - |
|  | E. coli | 57.1% | 100% | - | - | - | - | 0% | 0% | - | 71.4% | - | - | - | - |
|  | Acinetobacter | 66.6% | 66.6% | - | - | - | - | 16.6% | 0% | - | 66.66% | - | - | - | - |
| **[31]** | S. aureus | 7 (25.9%) | 13 (56.5%) | 11 (28.9%) | - | 7 (28.0%) | - | - | - | - | 13 (40.6%) | 25 (71.4%) | - | 3 (12.0%) | - |
|  | CoNS | 10 (22.2%) | 10 (47.6%) | 9 (20.9%) | 9 (20.9%) | - | 9 (22.5%) | - | 25 (83.3%) | - | 19 (44.2%) | 41 (91.1%) | - | 9  (100%) | - |
|  | Streptococcus | 12 (100%) | 7  (100%) | 14 (82.4%) | 11 (100%) | 10 (90.9%) | - | - | - | - | 1  (8.3%) | - | - | 2 (13.3%) | - |
|  | S. epidermidis | 4 (30.8%) | 7 (63.6%) | 4 (30.8%) | 4 (30.8%) | - | - | - | - | - | 7 (53.8%) | 6 (46.2%) | - | 1  (7.7%) | - |
|  | E. coli | 49 (35.3%) | 98 (79.0%) | 10 (9.6%) | 10 (9.8%) | 29 (22.3%) | - | - | - | - | 70 (50.7%) | 36 (25.9%) | - | 20 (15.5%) | - |
|  | Pseudomonas | 4 (50.0%) | 16 (84.2%) | 0  (0.0%) | 0  (0.0%) | 0  (0.0%) | - | - | - | - | 14 (60.9%) | 5 (55.6%) | - | 2  (9.5%) | - |
|  | Klebsiella | 7 (41.2%) | 11 (84.6%) | 2 (20.0%) | 2 (20.0%) | 4 (28.6%) | - | - | - | - | 12 (66.7%) | 5 (29.4%) | - | 7 (46.7%) | - |
|  | P. aeruginosa | 4 (50.0%) | 16 (84.2%) | 0  (0.0%) | 0  (0.0%) | 0  (0.0%) | - | - | - | - | 14 (60.9%) | 5 (55.6%) | - | 2  (9.5%) | - |
|  | Acinetobacter | 1 (14.3%) | 7 (87.5%) | 1 (16.7%) | 1 (16.7%) | 5  (100%) | - | - | - | - | 4 (44.4%) | 7 (77.8%) | - | 1 (20.0%) | - |
| **[18]** | Salmonella typhi | 25% | - | 17% | - | - | - | 21% | - | - | 31% | - | - | 18% | - |
| **[3]** | E. coli | 156 (21.2%) | 141 (28.8%) | ­- | - | - | - | - | - | - | 138 (30.3%) | - | 159 (19.7%) | - | 170 (14.1%) |
|  | K. pneumoniae | 16 (40.7%) | 10  (63%) | - | - | - | - | - | - | - | 13 (51.9%) | - | 23 (14.8%) | - | 25 (7.4%) |
|  | Proteus spp | NT | 4 (33.3%) | - | - | - | - | - | - | - | 4 (33.3%) | - | 5 (16.7%) | - | 5 (16.7%) |
|  | P. aeruginosa | 3  (75%) | 1  (91.7%) | - | - | - | - | - | - | - | 4 (66.7%) | - | 12  (0%) | - | 8 (33.3%) |
|  | S. aureus | 11 (15.4%) | 6 (57.1%) | 13 (7.1%) | 13 (7.1%) | - | - | - | - | - | 8 (42.9%) | - | 6 (57.1%) | - | - |
|  | MRSA | 11 (31.2%) | 11 (31.2%) | 14 (12.5%) | 10 (37.5%) | - | - | - | - | - | 13 (18.8%) | - | 11 (31.2%) | - | - |
|  | Staphylococcus | 6 (33.3%) | 6 (33.3%) | 9  (0%) | 9  (0%) | - | - | - | - | - | 5 (44.4%) | - | 6 (33.3%) | - | - |
|  | Enterococcus | 3 (62.5%) | 2  (75%) | NT | 8  (0%) | - | - | - | - | - | 2  (75%) | - | 4  (50%) | - | - |
| **[71]** | MRSA  (2015-2016) | - | 26 (43.33%) | - | - | - | - | 47 (78.33%) | 47 (78.33%) | - | - | 46 (76.66%) | - | 20 (33.33%) | - |
|  | MRSA  (2016-2017) | - | 61 (43.57%) | - | - | - | - | 97 (69.28%) | 83 (59.28%) | - | - | 106 (75.71%) | - | 19 (13.57%) | - |
| **[19]** | E. coli | 120 (27%) | 435 (99%) | - | - | 275 (63%) | - | - | - | - | 230 (52%) | - | 406 (92%) | - | - |
| **[73]** | A. baumannii | - | 15.4% | - | - | - | - | - | - | - | - | 44.2% | - | 21.8% | - |
| **[39]** | Acinetobacter | - | 29 (14.22%) | - | - | - | - | - | - | - | 13 (6.37%) | 63 (30.88%) | - | 14 (6.86%) | - |
| **[66]** | MRSA | - | 16  (0.0%) | - | - | - | - | 19  (63.5%) | 17  (0.0%) | - | 24  (0.0%) | - | N/A  (0) | 20  (68%) | - |
|  | MSSA | - | 0  (0.0%) | - | - | - | - | 33  (36.5%) | 0  (0.0%) | - | 0  (0.0%) | - | 145  (0.0%) | 42  (32%) | - |
| **[74]** | Gram positive | 108 (83.1%) | 112 (86.2%) | 6  (4.6%) | - | - | - | - | 104  (80%) | - | - | 117 (90%) | - | - | - |
|  | Gram negative | 39 (27.8%) | 116 (82.8%) | - | - | - | - | - |  | - | 72 (51.4%) | 62 (44.3%) | - | - | - |
| **[32]** | S. aureus | 75  (67%) | - | - | - | - | - | - | - | - | - | - | - | - | - |
|  | Streptococcus | 21 (61.8%) | - | - | - | - | - | - | - | - | - | - | - | - | - |
|  | MRSA | 0  (0.0%) | - | - | - | - | - | - | - | - | - | - | - | - | - |
|  | E. coli | 6 (27.3%) | - | - | - | - | - | - | - | - | - | - | - | - | - |
|  | Pseudomonas | 2 (14.3%) | - | - | - | - | - | - | - | - | - | - | - | - | - |
| **[20]** | E. coli | 34.8% | 52.2% | - | - | - | - | - | - | - | - | - | 43.5% | 8.6% | - |
|  | K. pneumoniae | 0.0% | 57.1% | - | - | - | - | - | - | - | - | - | 57.1% | 0.0% | - |
|  | P. aeruginosa | 66.7% | 100% | - | - | - | - | - | - | - | - | - | 0.0% | 33.3% | - |
| **[4]** | E. coli | 37.3% | 99% | 12.7% | - | - | - | - | - | - | 69% | - | 89.3% | 28.1% | - |
|  | Klebsiella | 0.0% | 75% | 0.0% | - | - | - | - | - | - | 56.2% | - | 29.4% | 47.1% | - |
|  | Enterobacter | 50% | 100% | 50% | - | - | - | - | - | - | 13.3% | - | 100% | 33.3% | - |
|  | Pseudomonas | 100% | 80% | 100% | - | - | - | - | - | - | 60% | - | 33.3% | 66.7% | - |
|  | Staphylococcus | 100% | 100% | 0.0% | - | - | - | - | - | - | 87.5% | - | 100% | 66.7% | - |
|  | Proteus | 98% | 66.7% | 0.0% | - | - | - | - | - | - | 66.7% | - | 0.0% | 75% | - |
|  | Citrobacter | 0.0% | 100% | 0.0% | - | - | - | - | - | - | 33.3% | - | 66.7% | 33.3% | - |
|  | Morganella | 0.0% | 100% | 0.0% | - | - | - | - | - | - | 100% | - | 0.0% | 100% | - |
| **[21]** | Salmonella typhi | - | - | 17% | - | - | - | 17% | - | - | - | - | - | 17% | - |
| **[70]** | A. baumannii | - | 12.6% | - | - | - | - | - | - | - | 13.3% | 45.9% | - | 19.5% | - |
| **[40]** | P. aeruginosa | - | 85.71% | 8.70% | 13.33% | - | - | - | - | - | 71.43% | - | - | 17.39% | - |
|  | K. pneumoniae | - | 69.57% | 4.55% | 66.67% | - | - | - | - | - | 66.67% | - | - | 34.78% | - |
|  | E. coli | - | 100% | 0% | 75% | - | - | - | - | - | 25% | - | - | 0% | - |
| **[46]** | CoNS  (n=39) | 15.8% | 86.1% | 8.1% | - | - | - | - | 51.3% | 38.9% | 30.3% | 92.3% | - | 34.3% | - |
|  | S. aureus  (n=29) | 21.4% | 69.2% | 4.2% | - | - | - | - | 59.3% | 41.4% | 38.5% | 96.4% | - | 74.1% | - |
|  | S. haemolyticus  (n=6) | 0.0% | 66.7% | 0.0% | - | - | - | - | 50% | 16.7% | 33.3% | - | - | 33.3% | - |
|  | E. faecium  (n=2) | 0.0% | NT | 0.0% | - | - | - | - | 0.0% | 0.0% | NT | 50% | - | 0.0% | - |
|  | K. pneumoniae  (n=18) | - | 25% | 0.0% | - | - | - | - | - | - | 20% | 37.5% | - | 14.3% | - |
|  | A. baumannii  (n=17) | - | 7.15% | 0.0% | - | - | - | - | - | - | 6.67% | 50% | - | 7.2% | - |
|  | P. aeruginosa  (n=9) | - | 44.5% | 0.0% | - | - | - | - | - | - | 33.4% | NT | - | 0.0% | - |
|  | E. coli  (n=6) | - | 40% | 0.0% | - | - | - | - | - | - | 25% | 75% | - | 0.0% | - |
|  | Serratia Marcescens  (n=4) | - | 75% | 0.0% | - | - | - | - | - | - | 50% | 66.67% | - | 50% | - |
| **[62]** | CR-AB isolates | - | 18.6% | - | - | - | - | - | - | - | - | 24.8% | - | 24.8% | - |
| **[5]** | E. coli | 2 (9.09%) | 21 (95.45%) | - | - | - | - | - | - | - | - | 5 (22.7%) | - | 4 (18.18%) | - |
|  | Klebsiella | 1 (33.33%) | 2 (66.66%) | - | - | - | - | - | - | - | - | 1 (33.33%) | - | 1 (33.33%) | - |
|  | Gram + cocci | 0 (0.0%) | 1 (50%) | - | - | - | - | - | - | - | - | 1 (50%) | - | 2 (100%) | - |
|  | Streptococcus | 0 (0.0%) | 1 (33.33%) | - | - | - | - | - | - | - | - | 1 (33.33%) | - | 2 (66.66%) | - |
|  | S. aureus | 2 (50%) | 3 (75%) | - | - | - | - | - | - | - | - | 0 (0.0%) | - | 2 (50%) | - |
|  | P. aeruginosa | 0 (0.0%) | 1 (100%) | - | - | - | - | - | - | - | - | 0 (0.0%) | - | 0 (0.0%) | - |
| **[68]** | CoPS | - | - | - | 7  (7.3%) | - | - | - | - | - | - | - | - | - | - |
|  | CoNS | - | - | - | 12  (20.4%) | - | - | - | - | - | - | - | - | - | - |
| **[33]** | S. aureus | 5 (18.52%) | 2 (7.41%) | - | - | - | - | 20 (74.07%) | - | 14 (51.85%) | 22 (81.48%) | 22 (81.48%) | - | 17 (62.96%) | - |
|  | E. coli | - | 20 (83.33%) | 2 (8.33%) | - | - | - | 4 (16.67%) | - | - | 16 (66.67%) | 7 (29.17%) | - | 9 (37.50%) | - |
|  | P. aeruginosa | - | 11 (61.11%) | - | - | - | - | - | - | - | 8 (44.44%) | - | - | - | - |
|  | MRSA | - | - | - | - | - | - | 20 (76.92%) | - | 1 (3.85%) | 10 (38.46%) | 18 (69.23%) | - | 12 (46.15%) | - |
|  | E. faecalis | - | 1 (14.29%) | 1 (14.29%) | - | - | - | 2 (28.57%) | - | - | 1 (14.29%) | 1 (14.29%) | - | 1 (14.29%) | - |
|  | K. pneumonia | - | 3  (60%) | - | - | - | - | - | - | - | 3  (60%) | 1  (20%) | - | - | - |
|  | A. baumannii | - | - | - | - | - | - | - | - | - | - | 3 (27.27%) | - | - | - |
|  | M. morganii | - | 1  (100%) | - | - | - | - | - | - | - | 1  (100%) | - | - | 1  (100%) | - |
|  | Bacillus subtilis | - | 1  (100%) | - | - | - | - | - | - | - | 1  (100%) | - | - | - | - |
|  | P. agglomerans | - | 3  (300%) | - | - | - | - | - | - | - | 2  (200%) | - | - | 2  (200%) | - |
|  | VRE | - | - | 1  (100%) | - | - | - | 1  (100%) | - | - | - | - | - | - | - |
| **[47]** | S. aureus  (n=24) | - | 88% | - | - | - | - | 84% | 55% | - | 50% | - | - | 67% | - |
|  | S. agalactiae  (n=6) | - | - | - | - | - | - | 17% | - | - | - | - | - | - | - |
|  | Klebsiella  (n=89) | 9% | 84% | 0.0% | - | - | - | 12% | - | - | 72% | - | 57% | 26% | - |
|  | E. coli  (n=98) | 13% | 88% | 17% | - | - | - | 42% | - | - | 56% | - | 88% | 20% | - |
|  | Serratia  (n=4) | 0.0% | 25% | 0.0% | - | - | - | 50% | - | - | 25% | - | 75% | 0.0% | - |
|  | Citrobacter  (n=4) | - | 25% | 0.0% | - | - | - | 50% | - | - | 75% | - | 100% | 0.0% | - |
|  | Proteus  (n=3) | 0.0% | 100% | 0.0% | - | - | - | 0.0% | - | - | 0.0% | - | 0.0% | - | - |
|  | A. baumannii (n=49) | - | 0.0% | - | - | - | - | - | - | - | 3% | - | - | 0.0% | - |
|  | P. aeruginosa  (n=51) | - | 88% | - | - | - | - | - | - | - | 82% | - | - | - | - |
|  | S. maltophilia  (n=5) | - | - | - | - | - | - | 20% | - | - | - | - | - | 20% | - |
| **[57]** | A. baumannii | - | 72.73% | - | - | - | - | - | - | - | 60% | - | - | 0.0% | - |
| **[24]** | E. coli | - | - | - | - | - | - | - | - | - | - | - | 78 (85.7%) | - | - |
| **[48]** | A. baumannii | 0  (0.0%) | 5  (2.5%) | 0  (0.0%) | - | - | - | 2  (1.0%) | - | - | - | - | - | 1  (0.5%) | - |
| **[63]** | CRACB isolates | - | 16.5% | - | - | - | - | - | - | - | - | - | - | - | - |
| **[67]** | S. aureus | 48  (12%) | 364 (91%) | 88  (22%) | - | - | 252 (63%) | - | 308  (77%) | - | 360 (90%) | - | 384 (96%) | - | - |
|  | S. pyogenes | 33  (33%) | 31 (61.5%) | 49  (98%) | - | - | 0 (0.0%) | - | 46  (92%) | - | 15  (30%) | - | 37  (74%) | - | - |
| **[59]** | P. aeruginosa | - | 29  (74%) | - | 0  (0%) | - | - | 14  (36%) | - | - | 7  (18%) | - | - | 6  (15%) | - |
| **[34]** | S. Aureus | - | - | 25 (41.7%) | - | - | - | - | - | - | 48  (80%) | - | - | 18  (30%) | - |
|  | MRSA | - | - | NT | - | - | - | - | - | - | NT | - | - | NT | - |
|  | Enterococcus | - | - | 3  (50%) | - | - | - | - | - | - | 5 (83.3%) | - | - | NT | - |
|  | B-streptococcus | - | - | 3  (50%) | - | - | - | - | - | - | 4 (66.7%) | - | - | NT | - |
|  | P. aeruginosa | - | - | NT | - | - | - | - | - | - | - | - | - | - | - |
|  | Acinetobacter | - | - | NT | - | - | - | - | - | - | - | - | - | - | - |
|  | E. coli | - | - | 15 (62.5%) | - | - | - | - | - | - | - | - | - | - | - |
|  | Klebsiella | - | - | NT | - | - | - | - | - | - | - | - | - | - | - |
| **[25]** | Klebsiella | 0% | 0% | 0% | - | - | - | - | - | - | - | 50% | - | 0% | - |
|  | E. cloacae | 0% | 100% | 0% | - | - | - | - | - | - | - | - | - | 100% | - |
|  | Salmonella typhi | 93% | - | 24% | - | - | - | - | - | - | - | 100% | - | 24% | - |
|  | Pseudomonas | - | 64% | - | - | - | - | - | - | - | 64% | - | - | - | - |
|  | Acinetobacter | - | 14% | - | - | - | - | - | - | - | - | 100% | - | 14% | - |
| **[26]** | E. coli | 50 (43.5%) | 80 (69.5%) | 79 (68.6%) | - | - | - | - | - | - | 35 (30.5%) | - | 69  (60%) | 70 (60.8%) | - |
| **[6]** | E. coli in Complicated UTI=47 | - | - | 4.5% | - | 24.6% | - | - | - | - | 55.8% | - | 79.5% | 18.9% | - |
|  | E. coli in Uncomplicated UTI=86 | - | - | 6.8% | - | 32.6% | - | - | - | - | 64.1% | - | 85.3% | 29.7% | - |
| **[7]** | H. parainfluenza | 57 (96.6%) | 58 (98.3%) | 52 (88.1%) | - | - | - | - | - | - | 59 (100%) | - | - | - | - |
|  | H. influenza | 65  (97%) | 54  (80.6 %) | 57 (85.1%) | - | - | - | - | - | - | 57 (85.1%) | - | - | - | - |
|  | S. pneumoniae | 30 (93.8%) | 24  (75%) | 27 (84.4%) | - | - | - | - | - | - | 28 (87.5%) | - | - | - | - |
| **[43]** | S. aureus | 52 (72.3%) | - | - | - | - | - | 61 (84.7%) | - | 51 (70.9%) | 72 (100%) | - | - | 40 (55.6%) | - |
|  | CoNS | 44 (57.2%) | - | - | - | - | - | 55 (71.5%) | - | 44 (57.2%) | 66 (85.8%) | - | - | 55 (72.5%) | - |
|  | S. pneumoniae | 11 (100%) | - | - | - | - | - | 11 (100%) | - | - | 11 (100%) | - | - | - | - |
| **[58]** | E. coli | - | 33 (80.5%) | 15 (36.6%) | - | - | 22 (53.7%) | - | - | - | 28 (68.3%) | - | - | 23 (56.1%) | - |
|  | P. aeruginosa | - | 93 (75.6%) | 17 (13.8%) | - | - | 10 (8.1%) | - | - | - | 30 (24.4%) | - | - | 26 (21.1%) | - |
| **[8]** | Enterobacteriaceae | 19.5% | 83.0% | 5.1% | - | - | - | - | - | - | 61.0% | - | 85.6% | 22.5% | - |
|  | Pseudomonas | - | 59.3% | - | - | - | - | - | - | - | 35.2% | - | - | - | - |
|  | Enterococcus | 54.4% | 28.6% | 55.2% | - | - | - | - | - | - | 33.3% | - | 75.3% | - | - |
|  | Staphylococci | 37.0% | 86.2% | - | - | - | - | - | - | - | 56.2% | - | 90.5% | 65.5% | - |
| **[27]** | E. coli | - | 77.3% | 13.3% | 9.3% | - | - | - | - | - | 64.7% | - | 70% | 28.7% | - |
| **[9]** | E. coli | - | 77% | 4.3% | - | - | - | - | - | - | - | - | 59.7% | - | - |
| **[49]** | Gram-positive isolates | 27.69% | 73.84% | - | - | - | - | - | - | - | 50.76% | - | - | 33.84% | - |
|  | Gram-negative isolates | 55% | - | - | - | - | - | 85% | - | - | 55% | 40% | - | 50% | - |
| **[28]** | E. coli | 2 (20%) | 6 (60%) | - | - | - | - | - | - | - | 6 (60%) | 6 (60%) | 6 (60%) | 0 (0.0%) | - |
|  | Klebsiella | 2 (50%) | 2 (50%) | - | - | - | - | - | - | - | 2 (50%) | 4 (100%) | 4 (100%) | NA | - |
|  | Enterobacter | NA | 0 (0.0%) | - | - | - | - | - | - | - | 0 (0.0%) | 2 (100%) | 2 (100%) | NA | - |
|  | Pseudomonas | 0 (0.0%) | 0 (0.0%) | - | - | - | - | - | - | - | 2 (100%) | 2 (100%) | 2 (100%) | NA | - |
| **[64]** | ESBL negative | 42 (56.8%) | 50 (67.6%) | - | - | - | - | - | - | - | 35 (47.3%) | 51 (68.9%) | - | 21 (28.4%) | - |
|  | ESBL positive | - | 21 (44.7%) | - | - | - | - | - | - | - | 3 (6.4%) | 37 (78.7%) | - | 27 (57.4%) | - |
| **[10]** | S. typhi | - | - | 0.0% | - | - | - | 27.6% | - | - | - | - | - | 1.1% | - |
| **[11]** | MRSA | 0% | 83.3% | 0% (IR) | - | - | - | 93.5% | 45.8% | - | 39.6% | 75% | - | 43.7% | - |
|  | MSSA | 100% | 100% | 3.4% | - | - | - | - | 79.3% | - | 68.9% | 58.6% | - | 55.2% | - |
|  | CoNS | 46.4% | 92.8% | 7.1% | - | - | - | - | 71.4% | - | 82.1% | 64.28% | - | 71.4% | - |
|  | E. faecalis | - | - | 80% | - | - | - | 80% | - | - | - | 25% | - | - | - |
|  | K. pneumoniae | 11.1% | 15.1% | 0% (IR) | - | - | - | - | - | - | 6.1% | 18.2% | - | 33.3% | - |
|  | A. baumannii | 0% (IR) | 10.5% | 0% (IR) | - | - | - | - | - | - | 15.8% | 47.3% | - | 31.6% | - |
|  | Stenotrophomonas maltophilia | 0% (IR) | 0% (IR) | - | - | - | - | - | - | - | 0% (IR) | - | - | 85.7% | - |
|  | P. aeruginosa | - | 33.3% | - | - | - | - | - | - | - | 33.3% | - | - | - | - |
|  | Enterobacter cloacae | 0% (IR) | 75% | 0% (IR) | - | - | - | - | - | - | 75% | 25% | - | 50% | - |
|  | K. oxytoca | 0% (IR) | 100% | 0% (IR) | - | - | - | - | - | - | 100% | 100% | - | 100% | - |
| **[12]** | E. coli | - | - | - | 77 (100%) | - | - | - | - | - | - | - | 58 (75.3%) | - | - |
|  | Klebsiella | - | 7  (100%) | 6 (85.7%) | 6 (85.7%) | - | - | - | - | - | 6 (85.7%) | - | 5 (71.4%) | 6 (85.7%) | - |
|  | Enterococcus | 3  (50%) | - | 3  (50%) | 3  (50%) | - | - | - | 0.0% | - | - | - | 5 (83.3%) | - | - |
| **[13]** | E. Coli | 26 (16.5%) | 140 (88.6%) | 10 (6.3%) | - | - | - | - | - | - | - | - | 134 (84.8%) | 19 (12.0%) | - |
|  | Klebsiella | 0  (0.0%) | 18 (18.6%) | 0  (0.0%) | - | - | - | - | - | - | - | - | 18 (18.6%) | 2  (2.1%) | - |
|  | Proteus | 2  (1.7%) | 20 (17.4%) | 0  (0.00%) | - | - | - | - | - | - | - | - | 20 (17.4%) | 2  (1.7%) | - |
|  | Pseudomonas | 0  (0.0%) | 8 (16.0%) | 0  (0.0%) | - | - | - | - | - | - | - | - | 8 (16.0%) | 0  (0.0%) | - |
|  | Enterobacteria | 2  (4.5%) | 8 (18.2%) | 1  (2.3%) | - | - | - | - | - | - | - | - | 9 (22.7%) | 2  (4.5%) | - |
|  | Acinetobacter | 0  (0.0%) | 4 (26.7%) | 0  (0.0%) | - | - | - | - | - | - | - | - | 1  (6.7%) | 0  (0.0%) | - |
|  | Citrobacter | 0  (0.0%) | 2 (28.6%) | 0  (0.0%) | - | - | - | - | - | - | - | - | 2 (28.6%) | 0  (0.0%) | - |
|  | Enterococcus | 2  (3.6%) | 6 (10.9%) | 1  (1.8%) | - | - | - | - | - | - | - | - | 6 (10.9%) | 1  (1.8%) | - |
|  | CoNS | 1  (0.4%) | 5 (15.4%) | 1  (0.4%) | - | - | - | - | - | - | - | - | 3  (7.7%) | 1  (0.4%) | - |
| **[69]** | S. aureus | - | - | 5% | - | - | - | - | 51% | - | 73% | - | 89% | 33% | - |
| **[14]** | Salmonella isolates | 25 (37.87%) | 30 (90.9%) | 1  (20%) | - | - | - | 6 (13.3%) | - | - | 22 (91.7%) | - | - | 11 (15.5%) | 1 (5.6%) |
| **[50]** | Pediatric cancer patients | 18  (6%) | 62  (21%) | 3  (1%) | 40  (13%) | - | - | - | - | - | 11  (4%) | - | 3  (0%) | 7  (2%) | - |
| **[35]** | S. aureus | 69 (67.6%) | - | - | - | - | - | - | - | - | - | - | - | - | - |
|  | Streptococcus | 19 (61.2%) | - | - | - | - | - | - | - | - | - | - | - | - | - |
|  | MRSA | 0  (0.0%) | - | - | - | - | - | - | - | - | - | - | - | - | - |
|  | E. coli | 5 (26.3%) | - | - | - | - | - | - | - | - | - | - | - | - | - |
|  | Pseudomonas | 2 (14.3%) | - | - | - | - | - | - | - | - | - | - | - | - | - |
| **[15]** | E. coli  (n=43) | 30.2% | 89.7% | - | 18.4% | - | - | - | - | - | - | - | 81% | 14% | - |
|  | Klebsiella  (n=11) | 36.4% | 100% | - | 0.0% | - | - | - | - | - | - | - | 70% | 18.2% | - |
|  | Enterococcus  (n=5) | 60% | NT | - | 40% | - | - | - | - | - | - | - | 100% | 0.0% | - |
|  | E. cloacae  (n=4) | 25% | 100% | - | 25% | - | - | - | - | - | - | - | 50% | 75% | - |
|  | P. aeruginosa  (n=4) | NT | 100% | - | NT | - | - | - | - | - | - | - | 0.0% | 0.0% | - |
|  | Proteus  (n=2) | 50% | 100% | - | 50% | - | - | - | - | - | - | - | 0.0% | 0.0% | - |
|  | S. saprophyticus  (n=2) | 100% | 100% | - | 50% | - | - | - | - | - | - | - | 100% | 50% | - |
| **[16]** | Uropathogens | (4%) | - | - | - | - | - | - | - | - | - | - | (99%) | (25%) | - |
| **[29]** | E. coli | - | 137 (74.4%) | 13 (7.06%) | 76 (41.3%) | - | - | - | - | - | 104 (56.5%) | 70  (38%) | - | 21 (11.4%) | - |
|  | P aeruginosa | - | 20 (25.6%) | 8 (10.5%) | 8 (10.2%) | - | - | - | - | - | 28 (35.8%) | 33 (42.3%) | - | 33 (42.3%) | - |
| **[51]** | E. coli | 11/74 (14%) | 80/93 (86%) | 6/94 (6.3%) | - | - | NA | 31/39 (79.4%) | 3/6 (50%) | - | 47/82 (55.2%) | - | - | 9/65 (13.8%) | - |
|  | P. aeruginosa | NA | 26/35 (74.2%) | NA | - | - | NA | 9/9 (100%) | NA | - | 14/30 (46.6%) | - | - | 1/9 (11.1%) | - |
|  | K. pneumoniae | 9/34 (36.4%) | 28/40 (70%) | NA | - | - | NA | 18/19 (94.7%) | NA | - | 16/31 (51.6%) | - | - | 4/12 (33.3%) | - |
|  | A. baumanni | NA | 9/26 (34.6%) | NA | - | - | NA | 4/10 (40%) | NA | - | 6/24 (25%) | - | - | NA | - |
|  | Proteus mirabilis | 3/20 (15%) | 15/27 (55.5%) | NA | - | - | NA | 6/16 (37.5%) | NA | - | 24/78 (43.5%) | - | - | 2/16 (12.5%) | - |
|  | MSSA | 8/10 (80%) | 101/112 (90.1%) | 6/29 (20.6%) | - | - | 21/35 (60%) | 81/86 (94.1%) | 97/125 (77.6%) | - | 82/108 (75.9%) | - | - | 17/79 (21.5%) | - |
| **[60]** | A. baumanni | - | 14 (10.77%) | - | - | - | - | - | - | - | 17 (13.08%) | 22 (16.92%) | - | 27 (20.77%) | - |
| **[52]** | E. coli  (n=32) | 15.62% | 75% | - | - | - | - | - | - | - | 75% | - | 78.12% | 15.62% | 15.62% |
|  | S. aureus  (n=17) | 47.05% | 36% | - | - | - | - | - | 41.17% | - | 82.35% | 58.82% | - | 18.75% | 41.17% |
|  | Klebsiella  (n=14) | 21.42% | 92.85% | - | - | - | - | - | - | - | 64.28% | - | - | 7.14% | 7.14% |
|  | Enterobacter  (n=10) | 20% | 80% | - | - | - | - | - | - | - | 60% | - | 60% | 60% | (60%) |
|  | Citrobacter  (n=9) | 33.33% | 7.77% | - | - | - | - | - | - | - | 66.67% | - | - | 55.55% | 44.44% |
|  | Acinetobacter  (n=6) | 0.0% | 0.0% | - | - | - | - | - | - | - | 33.33% | - | - | 0.0% | 0.0% |
|  | Pseudomonas  (n=5) | - | 66.67% | - | - | - | - | - | - | - | 66.67% | - | - | - | - |
|  | Proteus  (n=5) | 60% | 60% | - | - | - | - | - | - | - | 80% | - | - | 20% | 20% |
| **[23]** | E. coli  (n=132) | 28.7% | 21% | - | - | - | - | - | - | - | 67.3% | - | - | - | - |
| **[72]** | Salmonella typhi | - | 94 (90.38%) | - | - | - | - | - | - | - | - | - | - | - | - |
| **[17]** | Uropathogens | 0  (0.0%) | - | - | - | - | - | - | - | - | - | - | 17 (53.1%) | 12 (37.5%) | - |
| **[42]** | P. aeruginosa | 9 (14.51%) | 9 (14.51%) | - | - | - | - | 5 (8.06%) | - | - | 7 (11.29%) | - | - | 7 (11.29%) | - |
| **[56]** | E. coli | 12/86 (14%) | 87/103 (84.5%) | 7/104 (6.7%) | - | - | NT | 33/41 (80.5%) | NT | - | 51/96 (53.1%) | - | 13/14 (76.5%) | 12/81 (14.8%) | - |
|  | MSSA | 13/25 (52%) | 101/112 (90.2%) | 12/46 (26.1%) | - | - | 85.7% | 71/77 (92.2%) | 96/125 (76.8%) | - | 85/108 (78.7%) | - | NT | 21/83 (25.3%) | - |
| **[37]** | S. aureus  (n=50) | 2% | 36% | 1% | - | - | - | - | - | - | 38% | 34% | - | 16% | - |
|  | Streptococcus  (n=24) | 14% | 23% | 8% | - | - | - | - | - | - | 24% | 20% | - | 18% | - |
|  | P. aeruginosa  (n=32) | - | 21% | - | - | - | - | - | - | - | 17% | - | - | - | - |
|  | E. coli  (n=31) | - | 23% | - | - | - | - | - | - | - | 19% | - | - | - | - |
|  | Enterobacter  (n=21) | - | 13% | - | - | - | - | - | - | - | 16% | - | - | - | - |
| **[36]** | E. coli | 10.1% | 96.0% | - | - | - | - | 62.6% | - | - | - | 9.1% | - | 41.4% | - |
|  | P. aeruginosa | 54.70% | 83.70% | - | - | - | - | 43% | - | - | - | 0.0% | - | 0.0% | - |
| **[22]** | E. coli, Enterobacter and M. morganii | - | 78 (62.4%) | 19 (15.2%) | - | - | - | - | - | - | 63 (50.4%) | - | 41 (32.8%) | 47 (37.6%) | - |
| **[44]** | S. aureus  (hemodialysis) | - | - | - | - | - | - | - | - | - | 94.2% | - | - | - | - |
|  | CoNS  (hemodialysis) | - | - | - | - | - | - | - | - | - | 88.9% | - | - | - | - |
|  | E. coli  (hemodialysis) | - | - | - | - | - | - | - | - | - | 100% | - | - | - | - |
|  | K. pneumoniae  (hemodialysis) | - | - | - | - | - | - | - | - | - | 100% | - | - | - | - |
|  | P. aeruginosa  (hemodialysis) | - | - | - | - | - | - | - | - | - | 100% | - | - | - | - |
|  | Other gram-negative bacilli  (hemodialysis) | - | - | - | - | - | - | - | - | - | 93.3% | - | - | - | - |
| **[45]** | E. coli | 3.2% | 77.4% | 0% | - | - | - | - | - | - | - | 25% | - | 6.4% | - |
|  | K. pneumoniae | 0% | 75% | 0% | - | - | - | - | - | - | - | 0% | - | 5.2% | - |
|  | K. oxytoca | 0% | 100% | 0% | - | - | - | - | - | - | - | 50% | - | 75% | - |
|  | Serratia marcescens | 0% | 100% | 0% | - | - | - | - | - | - | - | 25% | - | 60% | - |
|  | Proteus mirabilis | 50% | 50% | 0% | - | - | - | - | - | - | - | ND | - | 100% | - |
|  | Enterobacter cloacae | 0% | 85.7% | 0% | - | - | - | - | - | - | - | 50% | - | 28.5% | - |
|  | Stenotrophomonas maltophilia | 0% | 0% | 0% | - | - | - | - | - | - | - | 66.6% | - | 100% | - |
|  | Burkholderia cepacian | ND | 0% | ND | - | - | - | - | - | - | - | ND | - | 50% | - |
|  | A. baumannii | 0% | 100% | 0% | - | - | - | - | - | - | - | 50% | - | 0% | - |
|  | P. aeruginosa | ND | 73.0% | ND | - | - | - | - | - | - | - | ND | - | ND | - |
|  | S. aureus | - | 57.14% | - | - | - | - | 100% | 71% | - | - | 57.14% | - | - | - |
|  | S. epidermidis | - | 54% | - | - | - | - | 100% | 57% | - | - | - | - | - | - |
| **[41]** | P. aeruginosa | 0  (0.0%) | 6 (23.08%) | - | - | - | - | - | - | - | - | - | - | - | - |
| **[53]** | A. baumannii | - | - | - | - | - | - | - | - | - | - | - | - | - | - |
|  | E. coli | 51/198 (25.8%) | - | 54/543 (9.9%) | - | 7/145 (4.8%) | 3/44 (6.8%) | - | - | - | - | - | - | - | - |
|  | K. pneumonia | 23/81 (28.4%) | - | - | - | 3/37 (8.1%) | 2/19 (10.5%) | - | - | - | - | - | - | - | - |
|  | Proteus spp. | 14/44 (31.8%) | - | 5/53 (9.4%) | - | 0/3 (0.0%) | 1/2 (50.0%) | - | - | - | - | - | - | - | - |
|  | P. aeruginosa | - | - | - | - | - | - | - | - | - | - | - | - | - | - |
|  | S. aureus | 27/48 (56.3%) | - | - | - | 60/138 (43.5%) | 35/67 (52.2%) | - | - | - | - | - | - | - | - |
| **[55]** | Acinetobacter | 1/36 (2.8%) | 10/36 (27.8%) | 1/35 (2.9%) | - | - | - | 9/35 (25.7%) | - | - | 10/36 (27.8%) | - | - | 9/35 (25.7%) | - |
|  | Citrobacter | 1/18 (5.6%) | 12/20 (60%) | 0/18 (0%) | - | - | - | 14/19 (73.7%) | 0/0 (0%) | - | 8/19 (42.1%) | - | - | 5/19 (26.3%) | - |
|  | E. coli | 3/67 (4.5%) | 55/68 (80.9%) | 0/67 (0%) | - | - | - | 57/68 (83.8%) | 0/0 (0%) | - | 38/68 (55.9%) | - | - | 13/68 (19.1%) | - |
|  | Klebsiella | 1/20 (5%) | 6/20 (30%) | 0/20 (0%) | - | - | - | 15/20 (75%) | 0/0 (0%) | - | 4/20 (20%) | - | - | 2/20 (10%) | - |
|  | Pseudomonas | 0/0 (0%) | 21/41 (51.2%) | 0/0 (0%) | - | - | - | 0/0 (0%) | 0/0 (0%) | - | 18/41 (43.9%) | - | - | 0/33 (0%) | - |
|  | Proteus | 4/30 (20%) | 22/30 (73.3%) | 2/30 (6.7%) | - | - | - | 7/30 (23.3%) | 0/0 (0%) | - | 14/30 (46.7%) | - | - | 3/30 (10%) | - |
|  | S. aureus | 0/0 (0%) | 147/175 (84%) | 0/0 (0%) | - | - | - | 168/172 (97.7%) | 128/174 (73.6%) | - | 119/174 (68.4%) | - | - | 63/177 (35.6%) | - |
|  | S. epidermidis | 0/0 (0%) | 8/11 (72.7%) | 0/0 (0%) | - | - | - | 9/11 (81.8%) | 7/11 (63.6%) | - | 3/11 (27.3%) | - | - | 4/11 (36.4%) | - |
|  | Streptococcus | 0/0 (0%) | 0/0 (0%) | 2/2 (100%) | - | - | - | 16/18 (88.9%) | 16/18 (33.3%) | - | 0/0 (0%) | - | - | 0/0 (0%) | - |
| **[54]** | Gram stain | 5 (8%) | 10 (17%) | 1 (1.7%) | - | - | - | - | 4 (7%) | - | 5 (8%) | 3 (5%) | - | 4 (7%) | - |
| **[38]** | S. aureus | - | - | - | - | - | - | - | - | - | - | - | - | - | 3 (30%) |
|  | P. aeruginosa | - | - | - | - | - | - | - | - | - | - | - | - | - | 2 (20%) |
|  | E. coli | - | - | - | - | - | - | - | - | - | - | - | - | - | 8 (80%) |
|  | K. pneumonia | - | - | - | - | - | - | - | - | - | - | - | - | - | 7 (70%) |
|  | C. perfringens | - | - | - | - | - | - | - | - | - | - | - | - | - | 9 (90%) |
| **[65]** | BCC isolates (n=134) | - | - | - | - | - | - | 103 (76.9%) | - | - | - | - | - | 69 (51.5%) | - |
|  | B. cepacia (n=92) | - | - | - | - | - | - | 68 (73.9%) | - | - | - | - | - | 48 (52.2%) | - |
|  | B. multivorans (n=25) | - | - | - | - | - | - | 20 (80%) | - | - | - | - | - | 11 (44%) | - |
|  | B. cenocepacia (n=17) | - | - | - | - | - | - | 15 (88.2%) | - | - | - | - | - | 10 (58.8%) | - |

**AMC**=augmentin (amoxicillin+ clavulanic acid/co-amoxiclav), **AMK**=amikacin, **AMP**=ampicillin, **AMX**=amoxicillin, **CEX**=cefalexin, **CFZ**=cefazolin, **CHL**=chloramphenicol, **CLI**=clindamycin, **CLOX**=cloxacillin, **CN**=gentamicin, **CoNS**=Coagulase negative staphylococcus, **DO**=doxycycline, **IMBB**=Institute of Molecular Biology and Biotechnology**, IR**=intrinsic resistance, **MIKD**=Multan Institute of Kidney Diseases Hospital**, NA**=not applicable, **ND**=not detected, **NT**=not tested**, NFT**=nitrofurantoin, **SXT**=sulfamethoxazole/trimethoprim (co-trimoxazole), **T**=trimethoprim.

**References:**

1. Yaseen, M., S. Rashid, and S. Naqvi, *Urinary tract infection in pregnant females attending antenatal clinics among middle socioeconomic settings.* The Professional Medical Journal, 2020. **27**(08): p. 1636-1641.

2. Shaikh, M., et al., *Spectrum and antimicrobial susceptibility pattern of micro-organisms associated with neonatal sepsis in a hospital in Karachi, Pakistan.* Cureus, 2020. **12**(10).

3. Muhammad, A., et al., *Prevalence and antibiotic susceptibility pattern of uropathogens in outpatients at a tertiary care hospital.* New Microbes and new infections, 2020. **36**: p. 100716.

4. Rizvi, Z.A., et al., *Exploring antimicrobial resistance in agents causing urinary tract infections at a tertiary care hospital in a developing country.* Cureus, 2020. **12**(8).

5. AHMAD, I., M.S. HUSSAIN, and M.S. AKHTAR, *Microbial spectrum and antibiotic sensitivity in cirrhotic patients with spontaneous bacterial peritonitis.*

6. Rana, M.A., et al., *Multidrug Resistant E-Coli in Patients with Urinary Tract Infections Presenting to Internal Medicine Clinics of Two Tertiary Care Hospitals in Lahore, Tip of the Iceberg.* Pakistan Journal of Medical & Health Sciences, 2022. **16**(02): p. 363-363.

7. Imdad, Z., et al., *Cultural Sensitivity of Sputum Bacteria Involved in Chronic Lung Disease and Type of Bacteria Involved in Chronic Lung Disease Sputum.* Pakistan Journal of Medical & Health Sciences, 2022. **16**(10): p. 702-702.

8. Idrees, M.M., et al., *A cross-sectional study to evaluate antimicrobial susceptibility of uropathogens from South Punjab, Pakistan.* Infection and Drug Resistance, 2022: p. 1845-1855.

9. Anwar, I., et al., *Antimicrobial Susceptibility Pattern of E. Coli in Patients with Urinary Tract Infection at a Tertiary Care Hospital, Rawalpindi.* Pakistan Journal of Medical & Health Sciences, 2022. **16**(06): p. 967-967.

10. Ali, A., N. Rahman, H. Adeeb, and I. Ullah, *Frequency and antimicrobial resistance profile of Salmonella Typhi isolated from District Buner.* Journal of Medical Sciences, 2022. **30**(3): p. 185-189.

11. Hussain, M.A., et al., *Neonatal Sepsis; Incidence and Microbiological Profile along with Antibiotic Sensitivity of Causative Microorganisms.* Life and Science, 2023. **4**(1): p. 6-6.

12. Karmani, J.K., A. Ahmad, M. Iqbal, and N. Arshad, *Antimicrobial Sensitivity Pattern of Urine Culture Isolate in a tertiary Care Hospital.* Pakistan Journal of Medical & Health Sciences, 2023. **17**(02): p. 61-61.

13. Jamal, M.M., et al., *Uropathogens and their Culture Sensitivity Pattern in Children with Urinary Tract Infection.* Pakistan Armed Forces Medical Journal, 2023. **73**(4): p. 1137-1140.

14. Sherazi, F., et al., *MULTIPLE DRUG RESISTANCE, EXTENSIVELY DRUG RESISTANCE TYPHOID FEVER AND DISEASE SPECTRUM IN PEDIATRIC POPULATION PRESENTING WITH FEVER WITHOUT LOCALIZING SIGNS (FWLS): A CROSS SECTIONAL STUDY.* KJMS, 2023. **16**(2): p. 103.

15. Iqbal, F., et al., *Bactericidal Effect of Antibiotics against Bacteria Causing Urinary Tract Infection among Children.* Pakistan Journal of Medical & Health Sciences, 2023. **17**(01): p. 824-824.

16. Zeb, S., et al., *PATTERN OF ANTIBIOTIC RESISTANCE IN STROKE PATIENTS WITH UTI AND ITS DETERMINANTS.* Journal of Population Therapeutics and Clinical Pharmacology, 2023. **30**(18): p. 989-994.

17. Ali, R., *Antibiotic susceptibility patterns of bacteria isolated from patients with community acquired urinary tract infections.* The Professional Medical Journal, 2023. **30**(11): p. 1501-1505.

18. Akhtar, S.T., et al., *Heading towards threat of resistant super bug…. current pattern of antimicrobial sensitivity to salmonella typhi in Karachi.* The Professional Medical Journal, 2020. **27**(05): p. 1070-1073.

19. Malik, J., et al., *Microbial resistance in urinary tract infections.* Cureus, 2020. **12**(5).

20. Khan, M.I., et al., *Assessment of multidrug resistance in bacterial isolates from urinary tract-infected patients.* Journal of Radiation Research and Applied Sciences, 2020. **13**(1): p. 267-275.

21. Durrani, A., *Emergence of Multi Drug Resistant Salmonella Typhi as Epidemic Among Lower Sindh Regions Patients of Pakistan: LMRJ.* LIAQUAT MEDICAL RESEARCH JOURNAL, 2020. **2**(4).

22. Shams, H., et al., *Extended Spectrum B-Lactamase (ESBL) and Metallo B-Lactamase (MBL) Production in Gram-Negative Bacteria isolated from Urinary Tract Infection Patients.* Journal of Saidu Medical College, Swat, 2023. **13**(2): p. 46-53.

23. Khan, N.B., et al., *A Study on Escherichia Coli Isolated from Urogenital Tract Infections, Emphasizing their Occurrence and Antibiograms.* Pakistan Journal of Medical & Health Sciences, 2023. **17**(03): p. 323-323.

24. Khan, H.A., et al., *Frequency of E coli and its sensitivity to nitrofurantion in patients with urinary tract infection.* Journal of Medical Sciences, 2021. **29**(02).

25. Raza, A., et al., *Prevalence and Antimicrobial Sensitivity Pattern of Gram Negative Rods in Blood Cultures: A Tertiary Care Hospital Study.* Pakistan Journal of Medical & Health Sciences, 2022. **16**(06): p. 124-124.

26. Noor, U., et al., *Prevalence and Phenotypic Detection of Carbapenem and Multi Drug Resistant of E. Coli in Urinary Tract Infection Patients in District Swat: Detection of Carbapenem and Multi Drug Resistant of E. coli.* Pakistan Journal of Health Sciences, 2022: p. 243-247.

27. Aqib, B. and R. Bilal, *Prevalence of multidrug-resistance of Escherichia coli in urinary tract infection patients visiting Rehman Medical Hospital, Peshawar, Pakistan.* 2022.

28. Afzal, E., S. Khan, M.K. Iqbal, and T. Ahmad, *Prevalence of urinary tract infection in children with cerebral palsy: Experience at tertiary care centre.* The Professional Medical Journal, 2022. **29**(12): p. 1866-1871.

29. Khan, M.R., et al., *Molecular Characterization and Epidemiology of Antibiotic Resistance Genes of β-Lactamase Producing Bacterial Pathogens Causing Septicemia from Tertiary Care Hospitals.* Antibiotics (Basel), 2023. **12**(3).

30. Fida, S., et al., *Clinical Perspectives of Multiple and Extensively Drug-Resistant Typhoid; result from a tertiary care hospital from Pakistan.* J Infect Dev Ctries, 2021. **15**(4): p. 530-537.

31. Sarwar, A., M.A. Butt, S. Hafeez, and M.Z. Danish, *Rapid emergence of antibacterial resistance by bacterial isolates from patients of gynecological infections in Punjab, Pakistan.* Journal of Infection and Public Health, 2020. **13**(12): p. 1972-1980.

32. Mahmood, T., et al., *Frequency of Common Pathogens Isolated from Open Fractures of the Extremities and their Antimicrobial Sensitivity Pattern.* Journal of Pakistan Orthopaedic Association, 2020. **32**(04): p. 197-201.

33. Ishtiaq, S. and I. Ahmed, *Susceptibility Pattern of Bacterial Isolates from Surgical Site Infections in a Tertiary Care Hospital at Rawalpindi.* Journal of Islamic International Medical College (JIIMC), 2021. **16**(4): p. 224-231.

34. Bhatti, R.A., et al., *Frequency of Various Causative Bacterial Organisims and their Culture Sensitivity Pattern in Patients with Open Tibial Fracture.* Pakistan Journal of Medical & Health Sciences, 2022. **16**(04): p. 217-217.

35. Hussain, A., et al., *Common Pathogen Frequency And Antimicrobial Sensitivity Pattern In Open Fractures Of The Extremities.* Journal of Pharmaceutical Negative Results, 2023: p. 174-180.

36. Abbas, G., H.A. Khan, S. Iqbal, and A. Nabi, *BACTERIAL ISOLATES AND THEIR SENSITIVITY PATTERNS IN PATIENTS WITH DIABETIC FOOT ULCERS.* Journal of Medical Sciences, 2023. **31**(01): p. 4-9.

37. Waheed, A., et al., *Bacteria causing community acquired superficial skin infections in tertiary care hospitals and their antibiotics susceptibility patterns.* Journal of Pakistan Association of Dermatologists, 2023. **33**(1): p. 108-115.

38. Aman, B.S., et al., *The bacterial profile and antibiotic susceptibility in skin and soft tissue infections at a tertiary care hospital of Quetta, Pakistan.* J Pak Med Assoc, 2024. **74**(7): p. 1249-1253.

39. Khurshid, M., et al., *In-Vitro Assessment Of The Therapeutic Potential Of Polymyxins And Tigecycline Against Multidrugresistant Acinetobacter Isolates From Infected Wounds.* J Ayub Med Coll Abbottabad, 2020. **32**(4): p. 459-464.

40. Maka, G., S. Shah, S. Bano, and S.A. Tunio, *Antibiotic susceptibility profiling of Gram-negative bacteria causing upper respiratory tract infections in Hyderabad, Sindh.* Journal of Life and Bio Sciences Research, 2020. **1**(01): p. 12-15.

41. Ullah, R., et al., *Presence of T3SS (exoS, exoT, exoU and exoY), susceptibility pattern and MIC of MDR-Pseudomonas aeruginosa from burn wounds.* J Infect Dev Ctries, 2023. **17**(8): p. 1130-1137.

42. Ahmad, J. and S. Ahmad, *Simultaneous Application Of Non-Antibiotics With Antibiotics For Enhanced Activity Against Multidrug Resistant Pseudomonas Aeruginosa.*

43. Mahmood, I., et al., *Frequency and Antibiotic Susceptibility of Bacteria Isolated in Patients with Blepharitis.* Pakistan Armed Forces Medical Journal, 2023. **73**(SUPPL-2): p. S338-41.

44. Gul, S., et al., *Catheter-related infections in Hemodialysis: Frequency and microbiological profile patients undergoing antimicrobial lock therapy with gentamicin for prophylaxis.* Biological and Clinical Sciences Research Journal, 2023. **2023**(1): p. 247-247.

45. Atif, M., *Isolation and Identification of Bacterial Pathogens Associated with Indwelling Devices and their Antimicrobial Susceptibility.* Tobacco Regulatory Science (TRS), 2023: p. 1177-1189.

46. Hussain, M., et al., *Bacterial spectrum and antimicrobial pattern of blood stream infections associated with non-tunneled double lumen catheter in hemodialysis patients.* Pakistan Armed Forces Medical Journal, 2021. **71**(4): p. 1161-66.

47. Zeshan, B., et al., *The Usage of Antibiotics by COVID-19 Patients with Comorbidities: The Risk of Increased Antimicrobial Resistance.* Antibiotics (Basel), 2021. **11**(1).

48. Fatima, K., et al., *Outbreak of Pan-resistant Acinetobacter Species in Intensive Care Units of a tertiary care hospital.* Pakistan Journal of Medical & Health Sciences, 2022. **16**(04): p. 43-43.

49. Anwar, Y., et al., *Increasing Antibiotic Resistant Pattern in Clinical Bacterial Isolates, From Tertiary Care Hospital, Hayatabad Medical Complex, Peshawar, Pakistan: Increasing Antibiotics Resistance in Hayat Abad Medical Complex.* Pakistan BioMedical Journal, 2022: p. 91-95.

50. Akhtar, A., et al., *Evaluation of Antibiotic Resistance in Pediatric Patients Suffering from Cancer.* 2023.

51. Altaf, U., et al., *Using Culture Sensitivity Reports to Optimize Antimicrobial Therapy: Findings and Implications of Antimicrobial Stewardship Activity in a Hospital in Pakistan.* Medicina (Kaunas), 2023. **59**(7).

52. Bashir, N., et al., *Antibiotics resistance as a major public health concern: A pharmaco-epidemiological study to evaluate prevalence and antibiotics susceptibility-resistance pattern of bacterial isolates from multiple teaching hospitals.* J Infect Public Health, 2023. **16 Suppl 1**: p. 61-68.

53. Saleem, Z., et al., *Antibiotic Susceptibility Surveillance in the Punjab Province of Pakistan: Findings and Implications.* Medicina (Kaunas), 2023. **59**(7).

54. Afzal, T., et al., *Antibiotic sensitivity pattern of deep skin and soft tissue infections in Pakistan.* The Professional Medical Journal, 2024. **31**(09): p. 1368-1374.

55. Khalid, N., et al., *Trends in antimicrobial susceptibility patterns of bacterial isolates in Lahore, Pakistan.* Front Antibiot, 2023. **2**: p. 1149408.

56. Haseeb, A., et al., *Impact of positive culture reports of E. coli or MSSA on de-escalation of antibiotic use in a teaching hospital in Pakistan and the implications.* Infection and Drug Resistance, 2023: p. 77-86.

57. Zahra, N., et al., *Phenotypic and genotypic evaluation of antibiotic resistance of Acinetobacter baumannii bacteria isolated from surgical intensive care unit patients in Pakistan.* Jundishapur Journal of Microbiology, 2021. **14**(4).

58. Hafeez, S.B., et al., *Catheter-Related Bloodstream Infection With Femoral Central Access Versus Internal Jugular Access in Patients Admitting to Medical Intensive Care Unit.* Cureus, 2022. **14**(9).

59. Kamran, M., et al., *Antimicrobial Susceptibility Pattern of Pseudomonas aeruginosa isolated from Clinical and Environmental Sources in Punjab, Pakistan: Antimicrobial Susceptibility Pattern of Pseudomonas Aeruginosa.* Pakistan BioMedical Journal, 2022: p. 34-38.

60. Taj, Z., et al., *Insights into the Intersection of Biocide Resistance, Efflux Pumps, and Sequence Types in Carbapenem-Resistant Acinetobacter baumannii: A Multicenter Study.* Pathogens, 2023. **12**(7).

61. Taj, S., *Antibacterial Susceptibility Pattern of Gram-Negative ESKAPE Pathogens*

*Isolated From Hospitalized Patients.* Journal of Islamic Internation Medical College, 2020. **15**(Volume.15 No. 4(2020): December).

62. Ejaz, H., et al., *Molecular epidemiology of extensively-drug resistant Acinetobacter baumannii sequence type 2 co-harboring bla NDM and bla OXA from clinical origin.* Infection and drug resistance, 2021: p. 1931-1939.

63. Ejaz, H., et al., *The Molecular Detection of Class B and Class D Carbapenemases in Clinical Strains of Acinetobacter calcoaceticus-baumannii Complex: The High Burden of Antibiotic Resistance and the Co-Existence of Carbapenemase Genes.* Antibiotics (Basel), 2022. **11**(9).

64. Kamran, M., et al., *Antibiogram And Prevalence Of ESBL Genes In Escherichia Coli From Clinical Specimen.* Blood, 2022. **23**: p. 19.0.

65. Saeed, M., et al., *Carbapenem-Resistant Burkholderia cepacia Complex Isolates Carrying bla (NDM-1) and bla (NDM-5) in Ventilator-Associated Pneumonia Patients and Contaminated Ventilator Tubing.* Transbound Emerg Dis, 2024. **2024**: p. 3352135.

66. Khan, M., et al., *Colonization and Antibiotic Resistance Profiling of Methicillin Resistant Staphylococcus Aureus (Mrsa) in Patients from Tertiary.* THE JOURNAL OF MICROBIOLOGY AND MOLECULAR GENETICS, 2020. **1**(3): p. 31-39.

67. Abbas, A. and S. Sarwar, *Antibiotic Susceptibility and Resistance of Clinical Isolates against Various Antibiotics: Antibiotic Susceptibility and Resistance.* MARKHOR (The Journal of Zoology), 2022: p. 29-32.

68. Hashmani, S., et al., *The resistance pattern of Staphylococci against beta-lactam group of antibiotics in Hyderabad, Pakistan.* Rawal Medical Journal, 2021. **46**(1).

69. Khalid, F., et al., *ANTIBIOGRAM OF STAPHYLOCOCCUS AUREUS AMONG CLINICAL ISOLATES AT A TERTIARY CARE HOSPITAL IN LAHORE.* Pakistan Postgraduate Medical Journal, 2023. **34**(03): p. 135-138.

70. Khurshid, M., et al., *Acinetobacter baumannii sequence types harboring genes encoding aminoglycoside modifying enzymes and 16SrRNA methylase; a multicenter study from Pakistan.* Infection and Drug Resistance, 2020: p. 2855-2862.

71. Ullah, A., et al., *METHICILLIN RESISTANT STAPHYLOCOCCUS AUREUS: ANTIBIOTIC RESISTANT TREND IN HAYATABAD MEDICAL COMPLEX PESHAWAR.* Annals of Allied Health Sciences, 2020. **6**(1).

72. Ahmad, A. and I.G. Afridi, *Antimicrobial sensitivity of salmonella typhi in children-A single center study.* The Professional Medical Journal, 2023. **30**(11): p. 1442-1444.

73. Khurshid, M., et al., *Dissemination of bla(OXA-23)-harbouring carbapenem-resistant Acinetobacter baumannii clones in Pakistan.* J Glob Antimicrob Resist, 2020. **21**: p. 357-362.

74. Jan, H., et al., *Causative organisms of surgical site infections and their antimicrobial susceptibility patterns in a general surgical ward in Peshawar”.* Pak J Surg, 2021. **37**(1): p. 9-13.
